# Tailored axillary surgery with or without axillary lymph node dissection followed by radiotherapy in patients with clinically node-positive breast cancer (TAXIS): study protocol for a multicenter, randomized phase-III trial

**DOI:** 10.1186/s13063-018-3021-9

**Published:** 2018-12-04

**Authors:** Guido Henke, Michael Knauer, Karin Ribi, Stefanie Hayoz, Marie-Aline Gérard, Thomas Ruhstaller, Daniel R. Zwahlen, Simone Muenst, Markus Ackerknecht, Hanne Hawle, Florian Fitzal, Michael Gnant, Zoltan Mátrai, Bettina Ballardini, Andreas Gyr, Christian Kurzeder, Walter P. Weber

**Affiliations:** 1 Department of Radiation Oncology, St. Gallen Cantonal Hospital, Rorschacher Strasse 95, 9007 St.Gallen, Switzerland; 2 Breast Center, St. Gallen Cantonal Hospital, Rorschacherstrasse 95, 9007 St. Gallen, Switzerland; 3 Department of Radiation Oncology, Graubünden Cantonal Hospital, Loestrasse 170, 7000 Chur, Switzerland; 4 Institute of Pathology, University Hospital Basel, Schönbeinstrasse 40, 4031 Basel, Switzerland; 5 Department of Biomedicine, University Hospital Basel, Hebelstrasse 20, 4031 Basel, Switzerland; 6 SAKK Coordinating Center, Effingerstrasse 33, 3008 Bern, Switzerland; 7 Department of Surgery, Medical University of Vienna, Währinger Gürtel 18-20, 1090 Vienna, Austria; 8 Department of Breast and Sarcoma Surgery, National Institute of Oncology, Ráth György u. 7-9, 1122 Budapest, Hungary; 9 Breast Unit, Gruppo MultiMedica, Via Fantoli 16/15, Milan, 20138 Italy; 10 Breast Center, University Hospital Basel, Spitalstrasse 21, 4031 Basel, Switzerland; 11 Faculty of Medicine, University of Basel, Klingelbergstrasse 61, 4056 Basel, Switzerland; 12 Breast Health Center, Comprehensive Cancer Center Vienna, Spitalgasse 23, 1090 Vienna, Austria; 13 IBCSG Coordinating Center, Effingerstrasse 40, 3008 Bern, Switzerland

**Keywords:** Breast cancer surgery, Axillary lymph node dissection, Clinically node-positive, Tailored axillary surgery, Axillary radiotherapy, Overall survival, Disease-free survival, Quality of life

## Abstract

**Background:**

Complete lymph node removal through conventional axillary dissection (ALND) has been standard treatment for breast cancer patients for almost a century. In the 1990s, however, and in parallel with the advent of the sentinel lymph node (SLN) procedure, ALND came under increasing scrutiny due to its association with significant patient morbidity. Several studies have since provided evidence to suggest omission of ALND, often in favor of axillary radiation, in selected clinically node-negative, SLN-positive patients, thus supporting the current trend in clinical practice. Clinically node-positive patients, by contrast, continue to undergo ALND in many cases, if only for the lack of studies re-assessing the indication for ALND in these patients. Hence, there is a need for a clinical trial to evaluate the optimal treatment for clinically node-positive breast cancer patients in terms of surgery and radiotherapy. The TAXIS trial is designed to fill this gap by examining in particular the value of tailored axillary surgery (TAS), a new technique for selectively removing positive lymph nodes.

**Methods:**

In this international, multicenter, phase-III, non-inferiority, randomized controlled trial (RCT), including 34 study sites from four different countries, we plan to randomize 1500 patients to either receive TAS followed by ALND and regional nodal irradiation excluding the dissected axilla, or receive TAS followed by regional nodal irradiation including the full axilla. All patients undergo adjuvant whole-breast irradiation after breast-conserving surgery and chest-wall irradiation after mastectomy. The main objective of the trial is to test the hypothesis that treatment with TAS and axillary radiotherapy is non-inferior to ALND in terms of disease-free survival of clinically node-positive breast cancer patients in the era of effective systemic therapy and extended regional nodal irradiation. The trial was activated on 31 July 2018 and the first patient was randomized on 7 August 2018.

**Discussion:**

Designed to test the hypothesis that TAS is non-inferior to ALND in terms of curing patients and preventing recurrences, yet is significantly superior in reducing patient morbidity, this trial may establish a new worldwide treatment standard in breast cancer surgery. If found to be non-inferior to standard treatment, TAS may significantly contribute to reduce morbidity in breast cancer patients by avoiding surgical overtreatment.

**Trial registration:**

ClinicalTrials.gov, ID: NCT03513614. Registered on 1 May 2018.

www.kofam.ch, ID: NCT03513614. Registered on 17 June 2018.

EudraCT No.: 2018–000372-14.

**Electronic supplementary material:**

The online version of this article (10.1186/s13063-018-3021-9) contains supplementary material, which is available to authorized users.

## Background

### Disease and therapy background

Worldwide more than 2 million patients are diagnosed with breast cancer every year [1]. It accounts for one third of all cancer diagnoses among women, and causes more than 600,000 deaths per year. Still about 1200 patients per year in Switzerland need axillary lymph node dissection (ALND) as part of their surgical treatment. ALND is indicated primarily for node-positive breast cancer. Patients are identified either by pre-operative evaluation of the axilla or by intraoperative sentinel lymph node biopsy (SLNB).

ALND has traditionally been considered standard care for all patients with breast cancer. Due to the morbidity of the procedure (ALND vs. SLNB Risk Ratio = 3.07 in a meta-analysis of 98 studies [2]) and decreasing axillary node involvement over time, a series of randomized controlled trials have been initiated in the 1990s and early 2000s to question this paradigm in patients with clinically negative axillary lymph nodes. These trials can be divided into four categories:

1. Omission of any surgical axillary staging in selected patients [3–6],
2. Omission of axillary dissection in all patients with negative sentinel lymph node (SLN) procedures [7–9]
3. Omission of axillary dissection in selected patients with limited nodal disease in the SLN [10–12]
4. Axillary radiation vs. axillary dissection [13–15] or observation [16]

The trial findings supported the current trend in clinical practice toward decreased rates of axillary dissection in patients with non-palpable axillary lymph node metastases [17, 18] and showed that axillary radiation is a valid alternative to dissection in selected patients.

The important landmark trial ACOSOG Z0011 has been criticized for several reasons. One limitation of ACOSOG Z0011 was the lack of standardization and detailed documentation of adjuvant radiation fields and the angles of the tangents, which makes it impossible to know how much of the axilla was irradiated [19]. Since the no-axillary dissection arm in Z0011 was categorized as “no further axillary treatment” and defined by “no axillary dissection and no third-field nodal irradiation,” this question became relevant. However, an attempt to reconstruct the radiation fields of Z0011 resulted in the receipt of only 30% of detailed radiotherapy (RT) records for centralized review, and produced evidence of improperly applied axilla irradiation in at least 20% of cases [20].

In parallel with the trend toward less axillary surgery, radiation oncologists have been broadening the indication for extended regional lymph node irradiation based on evidence from two large phase-III trials showing improved disease-free survival (DFS) [21–23] and a large population-based cohort study showing improved overall survival [24] in node-positive patients after ALND. In addition, recent data from the latest Early Breast Cancer Trialists’ Collaborative Group meta-analysis confirmed that post-mastectomy radiotherapy (PMRT) for patients with one to three positive nodes reduced recurrence and breast cancer mortality [25]. If given, PMRT includes the chest wall and regional nodes in most patients [26]. Consequently, the optimal contemporary loco-regional management of node-positive patients has become controversial, while ALND remains standard of care for patients with high-volume (i.e., large tumor load in the lymph nodes) or treatment-resistant nodal disease (i.e., residual disease in the lymph nodes after neoadjuvant systemic therapy).

### Ongoing trials

Two surgical trials have been initiated to provide evidence for the safety of omitting any axillary surgery in selected clinically node-negative patients (SOUND and INSEMA trials, please see Table 1 below).

**Table 1:** Ongoing clinical trials in axillary surgery [95]

	Country/name	Population	Randomization	Endpoint	Size	Start	End	TAXIS overlap
1	Italy: SOUND IEO S637/311 NCT02167490	cT1cN0 US negative	SLN vs. observation	DDFS	1560	Jan 2012	Jan 2017	No
2	Germany: INSEMA NCT02466737	cT1–2 cN0 US negative	1. SLN vs. observation 2. 1–2 SLN+ → ALND vs. no ALND	DFS	7095	Sept 2015	Sept 2024	No
3	France: SERC/IPC 2012–001 DNCT01717131	cT1–2 cN0	ALND vs. no ALND	DFS	3000	July 2012	July 2025	Minimal
4	China: Z0011-China NCT01796444	cT1–2 cN0 1–2SLN+	ALND vs. no ALND	DFS	Not shown	Jan 2013	June 2026	No
5	Sweden: SENOMAC NCT02240472	cT1–2 cN0 cT1–2 iN1 1–2 SLN+	ALND vs. no ALND	BCSS	3500	Jan 2015	Dec 2029	Minimal
6	United Kingdom: POSNOC NCT02401685	cT1–2 1–2 SLN+	ALND or axillary radiotherapy vs. no axillary treatment	Axillary recurrence	1900	Jan 2014	Mar 2023	No
7	Netherlands: BOOG 2013–07 NCT02112682	cT1–2 cN0 1–3 SLN+ mastectomy	ALND or axillary radiotherapy vs. no axillary treatment	RRR	878	June 2014	June 2027	No
8	USA: Alliance A011202 NCT01901094	cT1-3cN1 (S)LN+ after NACT	ALND+ extended regional nodal irradiation vs. axillary radiotherapy + extended regional nodal irradiation	IBC-RFI	2918	Feb 2014	Jan 2024	Partial

*ALND* axillary lymph node dissection, *BCSS*, *DFS* disease-free survival, *IBC*, *NACT*, *RFI*, *RRR*, *SLN* sentinel lymph node, *US* ultrasound

Several other ongoing randomized controlled trials primarily aim at validating the Z0011 protocol in different countries with several minor protocol modifications (Z0011-China, SERC/IPC 2012–001 in France, SENOMAC in Sweden, POSNOC in the United Kingdom, BOOG 2013–07 in the Netherlands).

POSNOC allows axillary RT as an alternative to ALND in the control arm. Similarly, BOOG 2013–07 allows axillary RT or ALND to complete axillary treatment in the control arm, but includes patients with one to three positive SLN undergoing mastectomy [27]. Since the Z0011 study population is not included in our proposed trial, there is no or only minimal overlap between these trials and the present protocol.

The most progressive ongoing clinical trial on axillary management, partially overlapping with the proposed protocol, is Alliance A011202. It compares ALND with axillary radiation in patients with residual disease after chemotherapy, which was an exclusion criterion in both the AMAROS and the Z0011 trials. The trial tests the hypothesis that the AMAROS protocol in combination with extended regional nodal irradiation works in these patients without ALND to the extent that chemotherapy-resistant lymph node metastases are as radiosensitive as chemotherapy-naive disease.

The optimal treatment of the regional lymph nodes in clinically node-positive patients is currently unclear, with less surgery, more RT or a combination thereof being under discussion. In the era of increasing post-mastectomy and extended regional node irradiation as well as effective systemic therapy, the indication for ALND in patients with clinically positive nodes and confirmed disease at the time of surgery has to be investigated in a clinical trial. Most of the above-mentioned trials excluded patients with residual metastases after neoadjuvant chemotherapy, and the omission of axillary dissection in patients with imaging-detected lymph node metastases is controversial since they may predict a larger volume of axillary disease [28–32].

### Rationale for performing the trial

TAXIS is designed to determine whether ALND may be no longer necessary for confirmed nodal disease at first diagnosis of breast cancer given the prevalence of extended regional nodal irradiation in clinically node-positive patients in the adjuvant setting or incomplete response of nodal disease in the neoadjuvant setting. In many patients undergoing ALND, the number of negative lymph nodes removed exceeds the number of tumor-affected nodes. Removing multiple unaffected lymph nodes increases morbidity with no therapeutic benefit. It is a consequence of the commitment of radical surgery to the principle of complete tissue removal within the anatomical borders of the axilla. Benefits and harms of this approach must be re-assessed. TAXIS investigates tailored axillary surgery (TAS), a tailored approach focusing on the removal of tumor-affected lymph nodes while limiting the extent of surgery to minimize the number of negative nodes removed. TAS may offer non-inferior oncological outcomes with less morbidity than ALND in patients with clinically positive nodes at first presentation and confirmed nodal disease at surgery with or without neoadjuvant therapy.

## Methods

### Trial design, sites, and duration

**Fig. 1:**
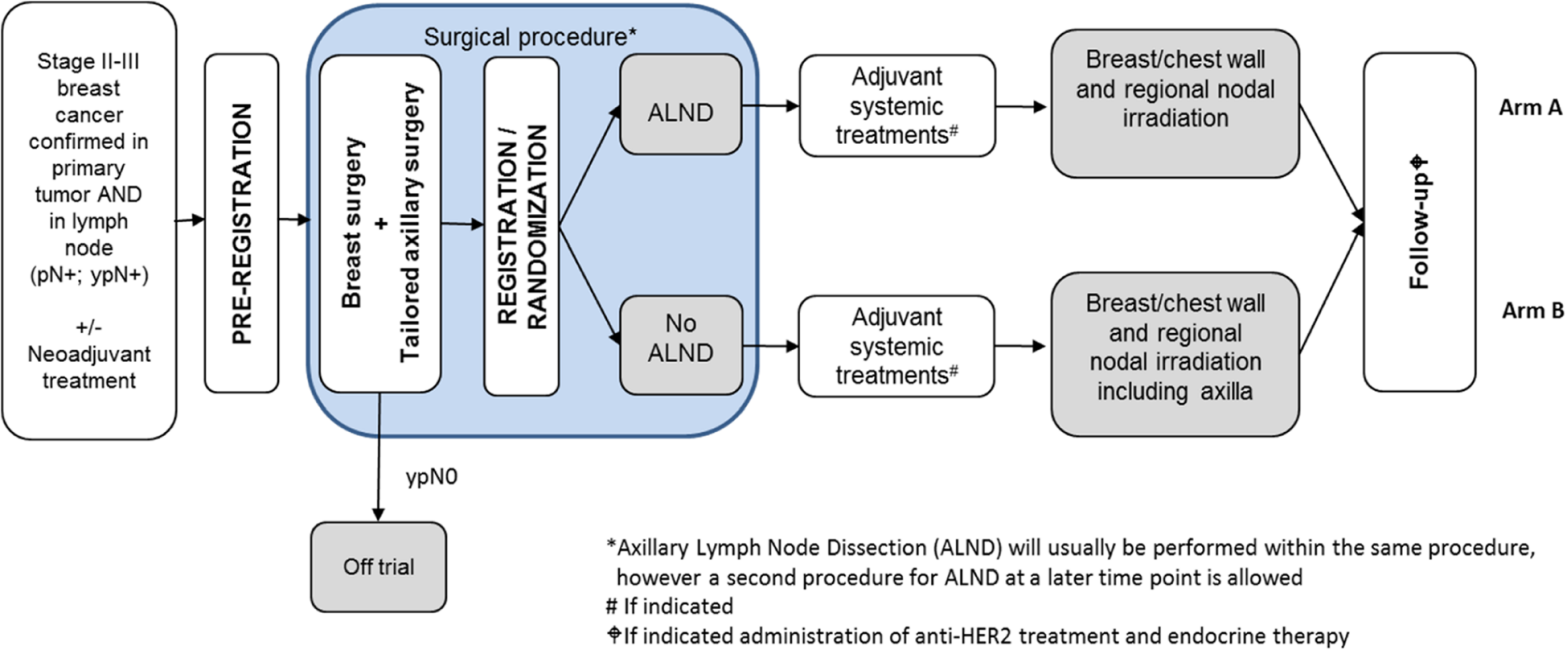
TAXIS trial flow chart

TAXIS is an international, multicenter, phase-III, non-inferiority randomized controlled trial with 34 study sites from four different countries currently planning to participate (Switzerland (Schweizerische Arbeitsgemeinschaft für Klinische Krebsforschung (SAKK)): 17; Austria (ABCSG): 10; Italy (IBCSG): 6; Hungary (IBCSG): (1). A list of sites and investigators participating in the trial can be downloaded from the public section of the SAKK website: http://sakk.ch/en/sakk-provides/our-trials/breast-cancer/sakk-2316-taxis/). Enrollment of patients started in July 2018 and will stop after the randomization of the targeted total of 1500 patients, which is expected in Q4 2023. End-of-trial treatment is expected for Q3 2024*.* All patients will be followed up for 20 years after randomization of the last patient. The trial will be terminated after the last visit of the last patient, which is expected to be in 2043*.* See Fig. 1 for the trial flow chart.

### Objective

The main objective of the trial is to test the hypothesis that treatment with TAS and axillary radiotherapy (RT) is non-inferior to axillary lymph node dissection (ALND) in terms of DFS of breast cancer patients with positive nodes at first presentation in the era of effective systemic therapy and extended regional nodal irradiation.

### Trial participants

#### Recruitment and consent

Prior to enrollment, eligible patients are informed about the aims, procedures and possible risks of the trial, as well as its confidentiality policy regarding patient data, and are given sufficient opportunity and time to consider whether or not to participate. All patients are made aware that participation is voluntary and that they are allowed to refuse further participation in the trial whenever they want. Written informed consent is obtained from each patient before enrollment and prior to any trial-specific procedures.

#### Inclusion criteria

Eligible for inclusion in this trial are female and male patients aged ≥ 18 years if they meet the following criteria:

Node-positive breast cancer (histologically or cytologically proven both in primary tumor and in lymph node) American Joint Committee on Cancer/Union for International Cancer Control (AJCC/UICC) [33] stage II–III (all molecular subtypes allowed), with or without neoadjuvant treatment planned

- Node-positivity detected by imaging (iN+) and confirmed by pathology or detected by palpation (cN1–2) and confirmed by pathology
- Node-positivity initially detected by imaging (negative on palpation) and residual disease intraoperatively confirmed by pathology (in SLN or non-SLN during surgery) in case of neoadjuvant treatment
- Node-positivity initially detected by palpation and residual disease intraoperatively confirmed by pathology in case of neoadjuvant treatment
- Eligible for primary ALND or SLN procedure with frozen section and either (1) newly diagnosed or (2) isolated in-breast recurrence or second ipsilateral breast cancer (at least 5 years disease free and no prior axillary surgery or loco-regional RT)
- Most suspicious axillary lymph node clipped
- Adequate condition for general anesthesia and breast cancer surgery
- Ability to understand and complete the quality of life (QoL) questionnaire
- WHO performance status 0–2

#### Exclusion criteria

Patients are excluded on any of the following grounds:

- Stage IV breast cancer
- Clinical N3 breast cancer
- Clinical N2 breast cancer (if limited to the internal mammary nodes only)
- Contralateral breast cancer
- Prior axillary surgery (except prior sentinel node procedure in case of in-breast recurrence)
- Prior regional RT
- History of hematological or primary solid tumor malignancy, unless in remission for at least 5 years from pre-registration with the exception of adequately treated cervical carcinoma in situ or localized non-melanoma skin cancer
- Concurrent treatment with any experimental drug within 30 days of pre-registration
- Concomitant use of other anticancer drugs or RT

Patients are, furthermore, excluded if they have any serious underlying medical, psychiatric, psychological, familial or geographical condition, which, in the judgment of the investigator, may interfere with the planned staging, treatment, and follow-up, affect patient compliance or place the patient at high risk from treatment-related complications.

### Randomization

A total of 1500 patients will be randomized at a ratio of 1:1 (750 patients per each treatment arm) to either receive TAS followed by ALND and regional nodal irradiation excluding the dissected axilla as a target volume (arm A), or receive TAS followed by regional nodal irradiation including the full axilla (arm B). Randomization is performed using the minimization method [34] with 80% allocation probability according to the following stratification factors:

- Responsible surgeon
- Type of positive node detection:
  - ○ Node-positivity detected by imaging (iN+) and confirmed by pathology
  - ○ Node-positivity detected by palpation (cN1–2) and confirmed by pathology
  - ○ Node-positivity initially detected by imaging (negative on palpation) and residual disease confirmed by pathology (in SLN or non-SLN during surgery) after neoadjuvant treatment
  - ○ Node-positivity initially detected by palpation and residual disease confirmed by pathology after neoadjuvant treatment
- Newly diagnosed vs. isolated in-breast recurrence or second ipsilateral breast cancer (at least 5 years disease free and no prior axillary surgery or loco-regional RT)
- Normofractionated vs. hypofractionated RT
- Male / female patient

Randomization is usually performed during the breast surgery as soon as the operating surgeon has certified that all inclusion criteria are met, including in particular the pathological confirmation of node-positivity and residual disease for patients who had received neoadjuvant treatment. If node-positivity cannot be confirmed on frozen section during primary surgery, the randomization takes place as soon as possible after the positive pathology results are available. Randomization is exclusively done online by using the centralized electronic data capture system secuTrial®. Patients who are not randomized will not enter into the trial and no further data will be collected except retrospective data for the two subprojects described in section “Performance characteristics of TAS” and “Patterns of use of neoadjuvant systemic treatment” of Appendix 3. Those patients will be treated according to the current best standard of care according to investigator’s decision, which commonly involves ALND.

### Investigational trial treatment

#### Overview

The investigational trial treatment consists of tailored axillary surgery (TAS), which is defined by the SLN procedure in combination with the selective removal of all palpable disease and documentation of the removal of the initially biopsy-proven and clipped lymph node metastasis by specimen radiography. If the clip is not documented in the specimen radiography, the patient is excluded and undergoes ALND.

All patients undergo adjuvant whole-breast irradiation after breast-conserving surgery and chest-wall irradiation after mastectomy. While patients allocated to arm A (control arm) receive regional nodal irradiation excluding the dissected axilla as a target volume (levels (II)/III; medial supraclavicular; internal mammary lymph nodes), patients allocated to arm B (investigational arm) receive regional nodal irradiation including the full axilla (levels I–III; medial supraclavicular; internal mammary lymph nodes).

If indicated, patients may undergo adjuvant systemic treatment.

#### Surgical procedure

##### Tailored axillary surgery

A video of the intervention has been uploaded as Additional file 1.


Additional file 1: Video S1. (MP4 383974 kb).


All patients undergo breast-conserving surgery or mastectomy and the SLN procedure. The SLN procedure should preferably be performed by dual mapping, which includes all nodes that are either blue (blue dye), hot (technetium Tc-99 m), fluorescent (indocyanine green) or magnetic (superparamagnetic iron oxide particles), according to local SLN procedure practices. In addition to the removal of the sentinel nodes, all palpably suspicious nodes, defined as either hardened or irregular or very large or a combination thereof, are removed.

Surgical evaluation of the levels I–III by palpation is mandatory to ensure that there is no palpably suspicious disease left behind in the axilla after TAS. The length of the incision and the opening of the clavipectoral fascia has to be chosen accordingly. Therefore, a minimum incision length of 2–3 cm is recommended. In case of palpably suspicious nodes left behind in the axilla after TAS, the patient must be excluded und typically undergoes ALND according to the decision of the treating surgeon.

Surgeons who perform the SLN procedure as part of their clinical routine are allowed to perform TAS. TAS is feasible for all surgeons who master the SLN procedure since the only difference is the selective removal of palpable disease, which we expect to be a frequent step in the clinically node-positive patient population of this trial. However, thorough palpation of the axilla is also mandatory during the regular SLN procedure in current clinical practice to remove suspicious findings and minimize the false-negative rate. The difference is that during the regular SLN procedure that is performed as a staging procedure, this step is rarely necessary since it is only performed in clinically node-negative patients.

The selective removal of the clipped lymph node by image-guided localization, a procedure increasingly referred to as targeted axillary dissection (TAD), is encouraged to increase the chances of successful clip removal. However, TAD is not a mandatory part of TAS, since it is technically challenging and, therefore, quite controversial, and the clipped lymph node corresponds to one of the SLNs in the majority of patients. TAD has recently emerged as an effective strategy to reduce the false-negative rate of the SLN procedure in patients with initially confirmed node-positive breast cancer that showed a complete clinical response in the nodes after neoadjuvant treatment. The metastatic node is marked with a clip during biopsy, or shortly after the lymph node metastasis has been confirmed by pathology or cytology, and is then selectively localized and removed during the procedure [35–37]. Any method is allowed for localization of the clipped node, such as the use of wire, iodine-125 or magnetic seeds, radioguided occult-lesion localization, ultrasound or a tattoo [35–41]. If the clip is not documented in the specimen radiography, the patient is excluded and undergoes ALND, since confirmation of the removal of the lymph node with the initially biopsy proven is not possible.

Importantly, residual suspicious lymph nodes detected by imaging before the end of adjuvant treatment does neither demand nor prohibit take-back surgery for completion ALND or selective removal of these nodes or an additional RT boost. The study group is fully aware that some patients will have non-palpable residual disease in the axilla after TAS. The hypothesis of TAXIS is that this residual disease does not progress to recurrence. The clinical scenario of residual disease after axillary surgery detected by imaging, i.e., performed for RT treatment planning or staging, to rule out distant disease, has become more frequent after implementation of the ACOSOG Z0011 and the EORTC-AMAROS protocols [13]. Histological evaluation and adjustment of axillary treatment (such as completion ALND, selective removal of suspicious nodes or additional RT boost) are allowed and interdisciplinary consensus-based decisions are encouraged.

##### Axillary lymph node dissection

After randomization, the patients in arm A will be treated according to the current standard of care by ALND. ALND is defined by the intention of the surgeon to radically remove the entire soft tissue within the anatomical borders of the axilla. To ensure applicability of the findings of the present study to clinical practice, we purposefully refrained from further defining the exact technique or number of removed lymph nodes. One common way to perform ALND is described here. Standard ALND clears levels I and II. A clip may be applied by the surgeon to mark the medial border of dissection, commonly between levels II and III, and documented in the surgical report. A full level-III dissection above the pectoralis minor muscle is carried out when there is gross nodal disease. The latissimus dorsi muscle is identified and followed until it is crossed by the axillary vein. The surface of the vein is then cleared of fat. Dissection inferior to the vein is carried out, dividing the fat and controlling the branches of the axillary vein entering the specimen. The thoracodorsal bundle is the deep lateral branch. Once identified, the pectoralis minor is retracted and the level-III nodes are dissected – if indicated – from the space below the axillary vein. The long thoracic nerve is identified against the chest wall and dissected free from the specimen. The fat between the thoracodorsal and long thoracic nerves is encircled with a clip or bipolar vessel-sealing system, divided, and bluntly swept inferiorly. Branches of the thoracodorsal vessels entering the specimen are dissected, and the specimen is freed from its remaining attachments to the inferior chest wall. A closed suction drain is standard care for most surgeons. The dermis is re-approximated and the skin is closed.

#### Radiotherapy

All patients undergo adjuvant whole-breast irradiation after breast-conserving surgery or chest-wall irradiation after mastectomy, as well as regional nodal irradiation, excluding the dissected axilla as a target volume in arm A (levels (II)/III; medial supraclavicular; internal mammary lymph nodes) and including the full axilla (levels I–III; medial supraclavicular; internal mammary lymph nodes) as a target volume in arm B. In case of extensive nodular involvement in arm A, the target volume includes the area at risk of the dissected levels. Inclusion of internal mammary nodes is recommended in this high-risk group of patients irrespective of treatment arm [42]. RT should start preferably within 8 weeks from the last breast surgical procedure and not later than 12 weeks. In case chemotherapy was applied, RT should start within 6 weeks after the end of the last cycle of chemotherapy and not later than 8 weeks. Dose to the breast/thoracic wall as well as the regional nodal pathways: 50 Gy in 25 fractions of 2 Gy or 50.4 Gy in 28 fractions of 1.8 Gy; daily, 5 days a week. Hypofractionated schedule allowed: 40 Gy in 15 fractions of 2.67 Gy to the same volume.

For a detailed description of RT, see Appendix 6.

#### Adjuvant systemic treatment

Where adjuvant systemic anticancer treatment is indicated, all drugs to be used are locally chosen according to international guidelines, including the sequence of systemic therapy in relation to surgery (neoadjuvant vs. adjuvant setting) [43, 44]. Handling of chemotherapy, anti-HER2 treatment and endocrine therapy is subject to the approved product information in each respective country. The decision of the indication of adjuvant chemotherapy could be different in the two treatment arms, because of a higher number of lymph nodes removed in arm A (ALND) compared to the group with TAS only. However, this belongs to a strategic trial like this and cannot be avoided with pre-defined rules in the protocol.

### Evaluations

For a description of all evaluations performed before, during, and after the trial treatment, see Appendix 1.

### Endpoints

#### Primary endpoint

The primary endpoint of the trial is disease-free survival (DFS), defined as time from randomization until one of the following events, whichever comes first:

- Local recurrence, regional recurrence, distant recurrence
- Second breast cancer
- Death from any cause

Patients not experiencing an event will be censored at the date of the last available assessment.

#### Secondary endpoints

The secondary endpoints of the trial are:

- QoL

The primary objective is to compare patient-reported arm problems in the short (after 9 months), intermediate (after 24 months), and long term (after 60 and 120 months) in breast cancer patients with clinically positive nodal disease in the era of extended regional nodal irradiation who are randomized to TAS followed by axillary radiation vs. TAS followed by ALND. The primary QoL endpoint is the change in the ARM subscale of the FACT-B + 4 from baseline to 24 months after randomization [45, 46].

Secondary objectives include the comparison of (1) short-, intermediate-, and long-term QoL and of (2) short-, intermediate-, and long-term effect of arm problems on daily and social activities between randomized groups.

For a detailed description of the QoL assessment procedure, see Appendix 2.

- Overall survival
- Breast cancer-specific survival
- Time to local recurrence
- Time to regional recurrence
- Time to distant recurrence
- Physician-reported morbidity outcomes
  - ○ Lymphedema: the change in ipsilateral upper-extremity circumference, corrected for any change in the contralateral upper extremity, will be calculated using the following formula:

$$ L=\left(I\mathrm{a}-I\mathrm{b}\right)-\left(C\mathrm{a}-C\mathrm{b}\right), $$

- where *I* indicates ipsilateral upper-extremity circumference, *C* indicates contralateral upper-extremity circumference, a indicates assessments during trial treatment and follow-up, and b indicates baseline assessment. *L* will be calculated for both upper arm and forearm, and lymphedema is defined as present if *L* > 2 cm for either location. The proportion of patients with lymphedema will be calculated at each assessment
- ○ Decreased range of shoulder motion: the change in shoulder motion (flexion, abduction, internal rotation, and external rotation) assessed by goniometric measurement of arm movement (Appendix 6) on both sides will be calculated for each assessment

Adverse events (AEs) according to National Cancer Institute Common Terminology Criteria for Adverse Events (NCI CTCAE) v4.03

Clipping-related AEs and specific AEs related to the surgical procedure and RT will be assessed according to NCI CTCAE v4.03

- Late RT-related AEs

Late AEs related to the RT will be assessed according to the Late Effects in Normal Tissues—Subjective, Objective, Management, and Analytic (LENT-SOMA) scale

- Surgical site infections (SSI)

SSI will be assessed according to the Centers for Disease Control and Prevention Surgical Site Infection Classification System.

### Subprojects

The following seven subprojects have been pre-specified:

1. Immune profile of axillary lymph nodes
2. Evaluation of the prognostic value of multigene tests
3. Performance characteristics of TAS
4. Patterns of use of neoadjuvant systemic treatment
5. Impact of TAS on adjuvant systemic treatment decisions
6. Quality assessment of delineation and dose planning and impact on treatment outcomes in adjuvant RT
7. Impact of a bolus on outcome and RT-related AEs in patients with mastectomy

For a detailed description of the subprojects, see Appendix 3.

### Sample size considerations and statistical analyses

The sample size is based on the primary endpoint DFS. With a type I error of 5% and a power of 80%, 385 events will be needed to show non-inferiority of TAS and axillary RT in comparison to ALND with a non-inferiority hazard ratio of 1.289 (corresponding to a DFS at 5 years of 80% in the ALND arm and 75% in the TAS and axillary RT arm). The sample size needed is 1500 patients (750 per arm). One interim analysis for efficacy/futility is foreseen after 20% of the required events have occurred. A report including the interim efficacy results as well as patient characteristics, treatment administration and safety results (AEs, SSI, and serious AEs) will be presented to an independent Data Monitoring Committee appointed by the SAKK Board. The SAKK Board will decide on the continuation/modification/early stopping of the trial based on the recommendations of the committee. All efficacy endpoints will be analyzed based on the per-protocol set. For the primary endpoint, DFS, the hazard ratio and one-sided 95% confidence interval will be calculated using a Cox regression model with the treatment arm as independent variable and the stratification factors as strata. The median DFS and the corresponding 95% confidence intervals using the Kaplan-Meier method will be presented for each treatment arm.

Planned subgroup analyses for the primary endpoint:

- Type of positive node detection at first diagnosis: cN1 vs. iN1
- Neoadjuvant vs. adjuvant systemic treatment vs. both
- ycN1 vs. ycN0
- BMI < 25 kg/m^2^ vs ≥ 25 kg/m^2^
- Normofractionation vs. hypofractionation
- Breast-conserving surgery vs. mastectomy
- Triple-negative vs. HER2+ vs. ER- and/or PR-positive, and HER2-

### Handling of missing data and drop-outs

No imputation of missing data will be performed. A row denoted “Missing” will be included in count tabulations if necessary to account for drop-outs and missing values. For continuous variables a column with the number of available observations will be added. Patients lost to follow-up before reaching the primary endpoint will not be replaced.

### Reporting of adverse events

Patients will be instructed by the investigator to report the occurrence of all AEs. The following events are reported by the investigator within the trial:

- Any AE related to the clipping of the most suspicious axillary lymph node and
- Specific AEs related to the trial surgical procedure or the RT (possible, probable, definite) as pre-defined in the original trial protocol

The investigator assesses and records the AEs observed during the AE-reporting period: from the date of patient consent signature up to 20 years after randomization of the last patient. However, no AEs related to neoadjuvant and/or adjuvant systemic therapy will be reported.

### Study management and administration

For a description of the study management and administration, see Appendix 4.

### Documentation

For a description of the study documentation, see Appendix 5.

## Discussion

Designed to test the hypothesis that TAS in combination with RT is non-inferior to ALND in terms of curing patients and preventing recurrences, yet is significantly superior in terms of patient morbidity, this trial has the potential to establish a new worldwide treatment standard in breast cancer surgery. If found to be non-inferior to standard treatment, TAS may contribute significantly to reduce morbidity in breast cancer patients by avoiding surgical overtreatment. For the SPIRIT checklist applicable to this study protocol, see Additional file 2.

## Trial status

Protocol no: SAKK 23/16 / IBCSG 57–18 / ABCSG-53

Protocol version: final version 1.0, 6 April 2018

EudraCT no: 2018–000372-14

Trial type: clinical trial with other health intervention

Categorization: risk category B according to the Swiss Human Research Act and its ordinance KlinV/Oclin

Trial sponsor: SAKK (Swiss Group for Clinical Cancer Research), Effingerstrasse 33, 3008 Bern, Switzerland

TAXIS was registered at ClinicalTrials.gov, on 1 May 2018, under NCT03513614 and on the Swiss National Clinical Trials Portal (SNCTP) at www.kofam.ch/de/studienportal/suche/38529/studie/42266, on 17 June 2018, under NCT03513614.

The trial was activated on 31 July 2018. The first patient was randomized on 7 August 2018.

### Additional files

Additional file 2: SPIRIT checklist. (DOCX 58 kb)

Additional file 3: QoL questionnaire. (PDF 514 kb)

## Figures and Tables

**Fig. 2:**
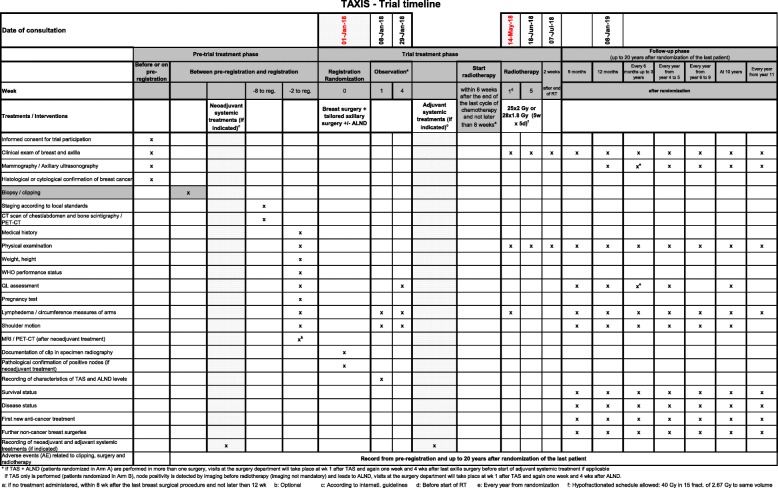
TAXIS trial timeline

## Appendix 1: Evaluations

### Evaluations before beginning of the trial treatment

- Clinical examination of the breast and axilla
- Mammography and axillary ultrasonography
- Histological or cytological confirmation of breast cancer in the primary tumor and in the lymph node
- Staging according to local standards (location of tumor within the breast, multicentricity/multifocality, type (no special type, lobular, other), grade, estrogen-receptor status, progesterone-receptor status, Her2 status, Ki-67 proliferation index)
- CT scan of chest/abdomen optionally in combination with bone scintigraphy / positron emission tomography-computed tomography (PET-CT)
- Medical history, including previous therapies, use of neoadjuvant systemic therapy, obesity, diabetes, smoking
- Physical examination, including weight and height and WHO performance status
- Completion of baseline QoL questionnaire
- Pregnancy test in blood or urine for women with child-bearing potential
- Baseline measurements of the circumference of both arms to assess potential lymphedema. This will be obtained 10 cm above and 5 cm below the olecranon process on both the ipsilateral and contralateral upper extremities
- Baseline evaluation of shoulder motion (flexion, abduction, internal rotation, and external rotation) on both sides will be assessed by goniometric measurement of arm movement

### Evaluations during surgical procedure

- Specimen radiography performed on all removed lymph nodes during TAS
- Pathological confirmation of positive nodes (SLN or non-SLN) prior to randomization in case of neoadjuvant treatment

### Evaluations after surgical procedure

If TAS ± ALND are performed in one surgery, visits at the surgery department take place at week 1 and 4 after TAS. If TAS + ALND (patients randomized in arm A) are performed in more than one surgery, visits to the surgery department take place at week 1 after TAS and again 1 week and 4 weeks after last axillary surgery before the start of adjuvant systemic treatment, if applicable. If TAS only is performed (patients randomized in arm B) and node-positivity is detected by imaging before RT (imaging not mandatory), and if this leads to ALND (not mandatory), then visits to the surgery department take place at week 1 after TAS and again 1 week and 4 weeks after ALND. If TAS + ALND are performed, node-positivity is detected by imaging before RT (imaging not mandatory) and leads to repeat ALND: no additional visit is required.

All AEs as pre-defined in the study protocol must be reported.

Occurrence of lymphedema: bilateral measurements will be taken at the sites assessed as at baseline. The measurements will be obtained 10 cm above and 5 cm below the olecranon process on both the ipsilateral and contralateral upper extremities.

Decreased range of shoulder motion: (flexion, abduction, internal rotation, and external rotation) on both sides will be assessed by goniometric measurement of arm movement*.*

Quality of life questionnaire: 4 weeks after TAS (TAS ± ALND in one surgery) or after last axillary surgery before start of adjuvant systemic treatment (TAS + ALND in more than one surgery).

Recording of characteristics of TAS and ALND levels (I–II vs. I–III).

Further investigations according to local standards.

### Evaluations during adjuvant systemic treatments (if indicated)

Visits at the Medical Oncology Department will take place according to the local practice.

Recording of all drugs used for adjuvant systemic anticancer treatment administered (if indicated)

### Evaluations during and after radiotherapy

- Symptom-guided physical examination
- Clinical examination of breast and axilla
- Adverse events

Before the beginning of RT only:

- Occurrence of lymphedema: bilateral measurements will be taken at the sites assessed at baseline. The measurements will be obtained 10 cm above and 5 cm below the olecranon process on both the ipsilateral and contralateral upper extremities
- Further investigations according to local standards

### Evaluations in the follow-up phase

The two first follow-up visits will take place 9 and 12 months after randomization then every 6 months up to 3 years, then every year up to 20 years after randomization of the last patient.

The patients will undergo the following assessments up to 10 years after randomization of the last patient unless they have been lost to follow-up, withdrawn consent or died.

Range of shoulder motion: (flexion, abduction, internal rotation, and external rotation) on both sides will be assessed by goniometric measurement of arm movement.

Quality of life questionnaire: to be filled in at 9 and 12 months after randomization then yearly during years 2 to 5 and at 10 years after randomization.

The patients will undergo the following assessments up to 20 years after randomization of the last patient unless they have been lost to follow-up, withdrawn consent or died:

- All AEs as pre-defined in the study protocol must be reported
- Occurrence of lymphedema: bilateral follow-up measurements will be taken at the sites assessed as at baseline. The measurements will be obtained 10 cm above and 5 cm below the olecranon process on both the ipsilateral and contralateral upper extremities
- Survival status
- Disease status
- Physical examination
- Clinical examination of breast and axilla
- Mammography and axillary ultrasonography every year from randomization
- First new anticancer treatment
- Further non-cancer breast surgeries

The coordinating investigator and the SAKK CC can decide to stop the follow-up period earlier. For a detailed timeline of the trial evaluations (see Fig. 2).

## Appendix 2: Quality of life

### Introduction

Breast surgery, including axillary dissection and adjuvant RT to the chest wall/breast and axillary lymph nodes, are frequently associated with impaired arm/shoulder function and development of lymphedema in up to 28% [54]. Patient-reported outcomes, including QoL, after ALND have been compared to those of SLNB in a number of studies and randomized trials. Differences were found with respect to subjective physical morbidity (e.g., pain, arm/shoulder morbidity), with worse symptom burden in those patients who had ALND [50–52, 55–57]. With respect to global QoL indicators, a meta-analysis of eight randomized trials found earlier recovery in QoL score for patients who had SLNB compared to ALND [58].

The long-term effect of ALND compared to SLNB on QoL has been studied with different lengths of follow-up periods and results are not consistent. While some studies suggest that the differences between the surgical procedures resolve over time (i.e., after 12 months) [50, 56, 57], others found that ALND results in poorer QoL than SLND over a longer period of 1 year [8], 2 years [59] or even 6 years [60].

In breast cancer survivors, regardless of type of local therapy, self-reported lymphedema, pain and restricted mobility in the arm/shoulder were significantly associated with poor long-term QoL [61]. The patient’s self-perception of physical morbidity and QoL is crucial because of the observation that patient self-ratings of arm swelling were generally more efficient than clinician’s measurement of arm volume in detecting differences between surgical groups [62] and to predict QoL [63]. Women without self-perceived arm lymphedema, regardless of objective arm lymphedema, reported better QoL in several domains than those who reported self-perceived arm lymphedema [64].

The current trial has the potential to offer non-inferior oncological outcomes while sparing many patients the relevant morbidity of ALND. Patient-reported outcomes, with respect to arm morbidity and QoL, is therefore, an important outcome for this trial.

### Objectives

The primary objective is to compare patient-reported arm problems in the short (after 9 months), intermediate (after 24 months), and long term (after 60 and 120 months) in breast cancer patients with clinically positive nodal disease in the era of extended regional nodal irradiation who are randomized to TAS followed by axillary radiation vs. TAS followed by ALND. The primary endpoint is the change in the ARM subscale of the FACT-B + 4 from baseline to 24 months after randomization.

Secondary objectives are:

- To compare short-, intermediate-, and long-term QoL in breast cancer patients with clinically positive nodal disease in the era of extended regional nodal irradiation who receive TAS followed by axillary radiation vs. TAS followed by ALND
- To compare the short-, intermediate-, and long-term effect of arm problems on daily and social activities in breast cancer patients with clinically positive nodal disease in the era of extended regional nodal irradiation who receive TAS followed by axillary radiation vs. TAS followed by ALND

### Patient selection

All patients are to complete the QoL questionnaire at the defined time points. Reasons for non-participation in the QoL study must be documented. To ensure compliance with the protocol requirements, the completion of the baseline QoL form is an eligibility criterion.

### Design

In order to evaluate intermediate- and long-term effects, a longitudinal design is used. The QoL questionnaire must be completed:

- At baseline (i.e., before surgical procedure)
- At 1 month after TAS (TAS ± ALND in one surgery) or after last axillary surgery before start of adjuvant systemic treatment (TAS + ALND in more than one surgery)
- At 9 and 12 months post randomization
- Yearly through years 2 to 5 (i.e., at months 24, 36, 48, and 60) post randomization
- At 10 years post randomization

### Assessment

Quality of life and patient-reported arm and shoulder problems will be assessed with the Functional Assessment of Cancer Therapy-Breast + 4 questionnaire (FACT-B + 4). The FACT-B is a well-validated measure of overall QoL specific to breast cancer [45]. It comprises five subscales: physical well-being (seven items), social well-being (seven items), emotional well-being (six items), functional well-being (seven items), and concerns specific to patients with breast cancer (13 items), that contains one item on arm morbidity. Four additional, validated, arm morbidity items were added to create an enhanced instrument, the FACT-B + 4 [46]. The arm functioning subscale comprises one item on arm morbidity included in the BCS and four additional items specific to arm morbidity. For a template of the QoL questionnaire in German, see Additional file 3.

#### Primary QoL endpoint

The ARM subscale (ARM) of the FACT-B + 4 will be used as the primary QoL endpoint. This subscale consists of five questions related to arm morbidity. Patients are asked to indicate on a 5-point scale from 0 (not at all), 1 (a little bit), 2 (somewhat), 3 (quite a bit), to 4 (very much), to what degree each statement applied over the previous 7 days.

The individual scoring of each question is inverted and added up to the total score ranging between 0 and 20 points, with higher scores representing fewer arm problems. Currently, there is no definition of minimally important differences (MID) for the FACT ARM subscale available. The ARM subscale has been shown to well discriminate between different surgical approaches, and the reference data from these two trials comparing SLNB (comparable to the intervention arm in this trial) with standard axillary dissection (corresponding to the standard arm in this trial) will be used for the interpretation of the clinical relevance of the findings in this trial [8, 56].

#### Secondary QoL endpoint

- All four FACT-B generic subscales (physical well-being, social/family well-being, emotional well-being, functional well-being)
- FACT-B breast cancer subscale (BCS)
- FACT-B trial outcome index (TOI: based on the physical, functional and breast-specific domains of the FACT-B)
- FACT-B total score

Scoring (FACT-B + 4 Scoring Guidelines (version 4) and interpretation of these subscales are based on standard definitions. A higher score indicates a better condition for all FACT subscales. Minimally important differences (MID) estimates were obtained; they are 2–3 points, 5–6 points, and 7–8 points for the BCS, TOI, and FACT-B, respectively [65].

The effect of breast-cancer-related arm problems on function and activity limitations and restrictions in social activities participation will be assessed by the Lymphedema Functioning, Disability, and Health questionnaire (Lymph-ICF) [66]. The 29-item questionnaire consists of five domains: physical function domain (seven questions); mental function domain (four questions); household domain (four questions); mobility domain (eight questions); and the life/ social life domain (six questions). Each question is scored on an 11-point scale (0 to 10). The anchor points for the physical and mental function domain, and household domain are “not at all” and “a lot.” Those for the mobility and life domain/ social life domain were “very well” and “not at all.” As recommended by the WHO taxonomy, the interpretation of the total score is as follows: 0–4 = “no problem,” 5–24 = “a small problem,” 25–49 = “a moderate problem,” 50–95 = “a severe problem,” 96–100 = “a very severe problem.”

### Statistical considerations

#### Submission rates

The submission rates of the QoL questionnaires will be calculated at yearly time points and will be used for trial monitoring. Rates below 70% and differences by the treatment arms indicate a problem and will require a review of the QoL assessment.

For the primary analysis, the available data on received questionnaires will be calculated for each item and scale.

#### Missingness

Reasons for missing data will be assessed for each scheduled assessment with no available QoL data and presented in frequency tables by treatment arm. In case of 20% or more missing questionnaires, the mechanism of missingness, i.e., missing completely at random (MCAR), missing at random (MAR) or missing not at random (MNAR), will be investigated and appropriate alternative analysis approaches might be applied.

#### Analyses

All endpoints will be analyzed based on the full analysis set. Supportive analyses based on the per-protocol set will be performed. The aim of all QoL analyses is to show superiority of the intervention arm compared to the standard arm.

Individual QoL scores will be descriptively summarized by treatment arm at each time point using median and range or mean and standard deviation.

For the primary QoL endpoint (FACT-B ARM subscale) the change from baseline to 24 months post randomization will be compared between treatment arms using linear regression models with the treatment arm as independent variable and the stratification factors as strata.

Subgroup analyses will be performed for patients with and without lymphedema during the first 24 months and by breast reconstruction.

Changes from baseline to the other time points will be analyzed in the same way.

The change in FACT-B ARM scale over the whole QoL assessment period will be compared between treatment arms using a repeated mixed model with the treatment arm as independent variable and the stratification factors as strata.

The secondary QoL endpoints will be analyzed in the same way as the primary QoL endpoint.

### Data management

#### Timing requirements

All QoL questionnaires are to be completed during the patients’ visits at the hospital at baseline, 1 month after TAS (TAS ± ALND in one surgery) or after last axilla surgery before start of adjuvant systemic treatment (TAS + ALND in more than one surgery), 9 and 12 months after randomization, then yearly during years 2 to 5 and 10 years after randomization. The schedule of the QoL assessment must be followed as closely as possible. A completed baseline questionnaire is an eligibility criterion and has, therefore, to be completed before registration. For the subsequent assessments, it is important that the QoL questionnaires are completed before any diagnostic procedures or communication of diagnostic or prognostic information to the patient, and before any treatment or supportive care measures.

#### Data collection and local data management

As part of the informed consent, the patients must be informed that there will be repeated assessments over 5 years and a final assessment 10 years after randomization.

All questions in the questionnaires must be answered. The completed QoL questionnaires are to be checked by the physician or research nurse while the patient is still present in the treating institution. If necessary, the patient should be asked to fill in missing answers.

Completed QoL questionnaires must be entered via the Internet (www.sakk.ch/edc) in a timely manner.

If a scheduled QoL assessment was not done, the reason has to be entered into the EDC system (see respective codes on the QoL questionnaire). Original QoL questionnaires are considered as source data and will be filed at the site.

Any questions regarding QoL or diary assessment may be addressed to Dr. Karin Ribi (karin.ribi@ibcsg.org or karin.ribi@sakk.ch).

## Appendix 3: Subprojects

### Immune profile of axillary lymph nodes

#### Description of the project

The immune profile of axillary lymph nodes in early stage breast cancer patients was previously shown to predict DFS [67]. However, whether such correlation exists at later stages is unknown. Furthermore, while the presence of metastatic cells in SLNs seems to be associated with a more immunosuppressed phenotype of the affected lymph node, it still remains controversial at which stage of the disease which immune cell subset is affected and to which extent [68–70]. The analysis of cancer cells and immune profiles of axillary lymph nodes, together with the assessment of matched primary tumor samples of the large cohort of patients enrolled in the present study will allow us to answer the following outstanding prognostic and basic questions in late-stage patients:

Basic short term questions:

- Do lymph node metastases preferentially grow at distinct sites (subcapsular sinus, cortex, medulla)? Which immune cells are present within, and in proximity to, the metastasis? Do these parameters correlate with characteristics of the primary tumor, the breast cancer subtype or neoadjuvant treatment?
- Does the immune profile of positive and negative lymph nodes correlate with characteristics of the primary tumor, the breast cancer subtype or neoadjuvant treatment?

Prognostic long-term questions:

c) Is there a correlation between DFS in advanced-stage breast cancer patients and the immune phenotype of positive and negative lymph nodes or the localization and infiltration of lymph node metastasis?

Depending on the processing of the lymph nodes and primary tumors right after dissections, i.e., formalin fixed or fresh, samples will be analyzed by immunohistochemistry and imaging mass cytometry of paraffin-embedded tissue sections or by ribonucleic acid (RNA) sequencing and flow cytometry of fresh samples, respectively. This approach will allow for a comprehensive analysis of several parameters (e.g., differentiation and activation state of T-cell subsets by CD4, CD8, IL-4, IL-10, IFNγ, FOXP3; maturation state of dendritic cells by CD1a, CD83, CD123, CD208; as well as analysis of neutrophils, macrophages, and NK cells) in order to clarify the impact of the metastatic disease on the immune phenotype of SLN and non-SLNs. Furthermore, these data will be correlated with clinical and non-clinical parameters of the primary tumor. Fresh primary tumor samples will also be maintained in mice as patient-derived xenografts (PDXs) which are subsequently used to test hypotheses generated based on the collected data.

After use, Professor M. Bentires-Alj will send the remaining paraffin block(s) and/or unstained section(s) for biobanking to:

Dr. Serenella Eppenberger-Castori

Biobank of University Hospital Basel

SAKK 23/16

Hebelstrasse 20,

CH-4031 Basel

Switzerland

After use, or in case of consent withdrawal, the PDXs will be sacrificed.

### Evaluation of the prognostic value of multigene tests

#### Description of the project

One of the main limitations of all multigene tests is that they are mainly validated to assess the risk of distant recurrence. However, a retrospective analysis of data from NSABP B14 + B20 suggested that the Oncotype DX® recurrence score (RS) may be prognostic for loco-regional recurrence (LRR) after mastectomy [71]. A retrospective analysis of data from the EORTC E2197 trial confirmed that the RS was not prognostic for LRR after breast-conserving surgery and radiation [72]. These findings have been validated in a third study of 163 patients that showed no association of RS with LRR after breast-conserving surgery and radiation. After mastectomy, however, the 5-year LRR rate was 27.3% in patients with a RS > 24 and 10.7% with a RS ≤ 24 (*p* = 0.04). The 70-gene profile also showed to be prognostic for LRR in a retrospective pooled analysis of 1053 patients. The 70-gene high-risk group had a LRR rate of 12.6% whereas the low-risk group had a rate of 6.1%. The 70-gene profile was an independent prognostic factor in this study [73]. The question of relevance is if PMRT can reduce the increased risk of LRR associated with high RS. The small number of patients with lRR after mastectomy makes this a difficult subject to study in a prospective trial; hence, there are currently no data to address this question. The present trial offers a great opportunity to study this question since this is a large, high-risk, node-positive patient population, and all patients will undergo chest-wall irradiation after mastectomy.

After use, the unstained section(s) will be destroyed and the remaining paraffin block(s) will be sent for biobanking to:

Dr. Serenella Eppenberger-Castori

Biobank of University Hospital Basel

SAKK 23/16

Hebelstrasse 20,

CH-4031 Basel

Switzerland

### Performance characteristics of TAS

#### Description of the project

The ACOSOG Z0011 and the EORTC AMAROS trials showed that in patients with known disease in the sentinel nodes that were selectively removed, axillary dissection does not further reduce the risk of axillary recurrence [12, 13]. In recent years, the sentinel procedure has gained increasing interest as a procedure to identify patients with complete pathological nodal response after neoadjuvant treatment [35, 74, 75]. The selective localization and removal of the clipped node has been shown to improve the accuracy of the sentinel procedure in this setting [36, 37]. Two prospective studies are currently validating these findings [76, 77].

TAXIS investigates the role of axillary dissection vs. axillary radiation in patients with confirmed and clipped nodal disease, detected by palpation or imaging. All patients undergo TAS before randomization. TAS is defined by combining the removal of the clipped node with the sentinel nodes and the palpably suspicious nodes. The removal of the clipped node is confirmed by specimen radiography. Selective localization of the clipped node with a wire, seed, tattoo or ultrasound is encouraged to increase the chances of successful removal of the clipped node. Selective localization of additional radiologically suspicious nodes is allowed (neither encouraged nor prohibited). In case of palpably suspicious nodes left behind after TAS, the patient is excluded. However, suspicious disease detected by imaging after surgery does neither demand nor prohibit take-back surgery.

#### Methods

This subproject prospectively evaluates TAS as a novel, less-invasive approach for treatment of the axilla in patients with confirmed nodal disease by combining the removal of the clipped and palpably suspicious nodes with the sentinel procedure with a focus on trends over time and by country.

#### Primary endpoint

First analysis after 200 randomized patients: rate of successful removal of clipped lymph node during tailored axillary surgery vs. without imaging-guided localization (targeted axillary dissection).

Analyses after 500 and 1500 randomized patients: rate of attempt to selectively localize the clipped node under imaging guidance (targeted axillary dissection) overall, over time during the trial period and by country.

#### Secondary endpoints

- Rate of successful localization of the clipped node when intended
- Imaging modality used to localize the clipped node (ultrasound, CT)
- Rate of successful removal of the clipped node as documented by specimen radiography in the group with successful localization
- Rate of successful removal of the clipped node as documented by specimen radiography in the group with intended but unsuccessful localization
- Rate of successful removal of the clipped node as documented by specimen radiography in the group without intended localization
- Number of failed identification and removal of sentinel nodes, defined as either radioactive or blue or both
- Type of clip used to mark the positive node (titanium or stainless steel marker with gel, ceramic marker with gel, titanium or stainless-steel marker, nitinol ring marker, direct radioactive seed, direct Magseed, other)
- Type of localization used to remove the clipped node (wire vs. seed vs. ROLL vs. tattoo vs. ultrasound)
  - ○ Number of sentinels removed during TAS (counted by the surgeon)
  - ○ Number of palpably suspicious findings removed during TAS (counted by the surgeon)
  - ○ Number of positive nodes removed by TAS (counted by the pathologist)
  - ○ Number of positive nodes removed by ALND after TAS (counted by pathologist)
  - ○ Number of negative nodes removed by TAS (counted by pathologist)
  - ○ Number of patients taken back for surgery before start of RT for residual disease suspected by imaging
  - ○ Number of patients receiving an extra radiation boost for residual disease suspected by imaging

#### Pre-specified analyses

This pre-specified subproject plans to analyze the performance characteristics of TAS after the first 200, 500, and after completed recruitment of 1500 randomized patients. Furthermore, the patients pre-registered but not randomized will also be evaluated for this subproject. Full analysis details will be provided in a statistical analysis plan.

### Patterns of use of neoadjuvant systemic treatment

Randomized controlled trials have not shown a significant difference in survival outcomes in patients undergoing the neoadjuvant vs. the adjuvant chemotherapy regimen [78]. However, due to several advantages, such as de-escalation of surgery and the prognostic information based on response to treatment, the neoadjuvant approach seems to be increasingly used [43, 79]. TAXIS is unique inasmuch as it allows both the neoadjuvant and adjuvant regimens according to the preference of the treating physicians and thus provides an excellent opportunity to study patterns of use of neoadjuvant treatment in patients with clinically positive nodes over time and by country.

Hypothesis: there is clinical practice heterogeneity in the use of neoadjuvant treatment in Europe, which is decreasing over time.

#### Primary endpoint

First analysis after 500 randomized patients: rate of patients undergoing neoadjuvant treatment.

Second analysis after completed recruitment of 1500 randomized patients: rate of patients undergoing neoadjuvant treatment over time.

#### Secondary endpoints

- Rate of patients undergoing neoadjuvant treatment by country, by site, and by surgeon
- Rate of patients undergoing neoadjuvant treatment by stage
- Rate of patients undergoing neoadjuvant treatment by intrinsic subtype, defined by the expression of hormonal receptors and human epidermal growth factor receptor 2 (HER2)
- Proportion of patients undergoing neoadjuvant treatment with pathological complete response in axillary nodes by intrinsic subtype

#### Pre-specified analyses

This pre-specified subproject plans to analyze patterns of care in the use of neoadjuvant treatment after the first 500 randomized patients and after completed recruitment of 1500 randomized patients. Furthermore, the patients pre-registered but not randomized will also be evaluated for this subproject. Full analysis details will be provided in a statistical analysis plan.

### Impact of TAS on adjuvant systemic treatment decisions

Today, the indication for chemotherapy is primarily based on tumor biology, with most patients with a triple-negative or Her2-positive breast cancer receiving some sort of chemotherapy. In patients with luminal breast cancers, particularly luminal A cancer, the indication for chemotherapy still depends on the total number of positive nodes, with four or more positive nodes implying a relative indication for the vast majority of the 2015 St. Gallen Consensus Conference Panel. However, the lack of knowledge of the exact number of positive nodes did not increase the likelihood of patients in the SLN-only arm of both the ACOSOG Z0011 and EORTC AMAROS trials to receive chemotherapy [12, 13]. Hypothesis: the lack of the exact number of positive nodes provided by TAS compared to ALND does not have an impact on adjuvant systemic treatment decisions in patients with confirmed nodal disease at primary diagnosis.

#### Primary endpoint

Rate of patients undergoing chemotherapy after TAS vs. ALND

#### Secondary endpoints

- Differences in type of chemotherapy after TAS vs. ALND
- Differences in type of endocrine therapy after TAS vs. ALND
- Differences in length of endocrine therapy after TAS vs. ALND

#### Pre-specified analyses

This pre-specified subproject plans to analyze the impact of TAS on adjuvant systemic treatment decisions after the first 500 and after completed recruitment of 1500 randomized patients. Full analysis details will be provided in a statistical analysis plan.

### Quality assessment of delineation and dose planning and impact on treatment outcomes in adjuvant radiotherapy

#### Background

Adjuvant and loco-regional RT offers benefit in terms of local control and DFS after mastectomy and lumpectomy in early breast cancer and is offered to an increasing number of patients. On the other hand, morbidity, such as radiation dermatitis, lymphedema, impaired shoulder motion, pneumonitis or cardiac events, are related to extended treatment and, therefore, optimal RT is essential to obtain the best effect at the lowest risk of morbidity.

In modern RT this is achieved by employing a target-volume-based approach in contrast to a field-based treatment used in the previous decades. The appropriate dose to the target volume can individually be calculated independent of the applied technique. This allows broad participation of RT institutes and accrual of a large and representative sample of patients treated with different and complex techniques including 3D-CRT techniques or IMRT as well as rotational techniques, such as Tomotherapy®, RapidArc®, IMAT or VMAT, to achieve best protocol-compliant target coverage as well as sparing of organs at risk (OAR).

It is well recognized that deviations from protocol guidelines can have an adverse effect on the results of a trial. Therefore, quality assessment of a multicenter trial is crucial to ensure uniformity of the treatment especially when different fractionations and techniques are used and the target volumes differ between the treatment arms like in the TAXIS trial.

#### Objectives

Since delineation in breast cancer treatment planning may be prone to large inter-observer variability and, nevertheless, is the basis of modern treatment planning, the agreement of target delineation with the protocol guidelines should be prospectively assessed.

The dose-volume histograms of the breast/chest wall, the distinct lymph node levels and the OAR, as well as other prospectively defined treatment parameters, should be evaluated with regard to the applied technique and the treatment arm.

Furthermore, the potential effect of dose coverage and these treatment parameters on survival outcomes, treatment-related toxicity and morbidity stratified by treatment arm and type of surgery, should be analyzed prospectively with regard to the applied radiation technique, fractionation schedule, use of a bolus after mastectomy and the application of a boost to the tumor bed.

#### Methods

1. Quality assurance
  - DICOM-RT files, with structure set and the dose distribution of a dummy run for both treatment arms conducted before initiation of a participating site, and of individual case reviews (ICRs) for the first three patients in each treatment arm in every site, will be submitted by the participating sites
  - Qualitative assessment (per protocol; minor deviation; major deviation) of compliance with the protocol by two independent reviewers for the dummy run (DR) and the ICRs. Hypotheses: major deviations should not occur in more than 10% of reviewed cases
  - Calculation of the dice similarity coefficient for the DR to evaluate the overlap between the original delineation of the participating sites and a centrally approved re-delineation according to the EORTC guideline. Assessment of time dependency
  - DVH comparison of the ICR treatment plans for the original target structures and the re-delineated target structure. Parameters to be analyzed are Dmin, Dmax, Dmean, D50%, D95%, V85%, and V50% for the clinical target volumes (CTVs) and the nodal areas
2. Impact of treatment parameters on toxicity, morbidity, and outcome parameters (all patients with complete RT CRFs according to Appendix 6, section “Quality assurance of radiotherapy”)
  - Evaluation of the following RT-relevant parameters: CTV DVH parameters, nodal DVH parameters, treatment interruption, OAR DVH parameters, use of a bolus, type of breast surgery
  - Evaluation of the RT-related AEs will take place at the end of the RT, 2 weeks after RT as well as during long-term follow-up

#### Statistics

Statistical evaluation will be performed at the SAKK Coordinating Center. Exploratory uni- and multivariable analyses for normal tissue and tumor-specific DVH parameters will be performed with acute and late-RT-related AEs, DFS, time to local recurrence, time to regional recurrence and overall survival as endpoints.

Full analysis details will be provided in a statistical analysis plan.

### Impact of a bolus on outcome and RT-related adverse events in patients with mastectomy

#### Background

Post-mastectomy radiotherapy (PMRT) is indicated in patients with higher risk for loco-regional failure. In this trial, all patients with mastectomy will receive irradiation of the chest wall. PMRT can be given with or without a tissue-equivalent bolus on the skin surface. The purpose of a bolus is to increase the radiation dose on the skin [80]. The additional benefit of using a bolus remains unclear. Vu et al. did an international survey: the respondents showed an extensive use in the USA (82% of clinicians always using a bolus), and a less frequent use in Europe (31%) and Australasia (65%) [81]. No established consensus exists, which is mainly due to the lack of evidence regarding the efficacy of a tissue bolus in reducing chest-wall recurrence [82]. However, it is well known that use of a bolus is associated with a higher incidence of skin toxicity with greater acute toxicity (e.g., moist desquamation) and more treatment interruptions [83] and late effects including skin telangiectasia [84]. Published data are mainly based on rather small, retrospective cohort studies.

To our knowledge, this subproject would be the largest, prospectively generated analysis on the use of a bolus in PMRT.

#### Hypothesis

The use of a bolus will result in a better CTV coverage of the chest wall, but will not influence TTLR and DFS. On the other hand, the use of a bolus will increase skin toxicity and reduce QoL.

#### Methods and statistical considerations

With an estimated mastectomy rate of about one third, about 500 patients will be available for analyses. Descriptive analyses about the use of a bolus will be performed in regard to country, type of surgery (mastectomy with/without immediate reconstruction) and other patient-, tumor-, and treatment-related parameters. This subproject will compare the impact of a bolus on local recurrence and DFS as well as on skin toxicity, treatment interruptions and QoL by exploratory uni- and multivariable analyses. In addition, correlations are planned for CTV coverage (see also Appendix 6, section “Quality assurance of radiotherapy”). Statistical evaluations will be performed at the SAKK coordinating center.

## Appendix 4: Study management and administration

### Monitoring and auditing

All source data are accessible for auditing and monitoring. Clinical research associates (CRAs) and auditors will maintain patient confidentiality. The trial is being monitored. The SAKK is following a risk-adapted monitoring strategy developed according to the concept developed by the ADAMON Group [85] and the TransCelerate position paper [86]. The different monitoring activities, as well as the frequency of the visit, are described in a trial-specific monitoring plan based on the risk analysis. Authorities have the right to perform inspections, and the SAKK has the right to perform on-site auditing during working hours upon reasonable prior notice.

### Independent Data Monitoring Committee

An independent Data Monitoring Committee appointed by the SAKK Board evaluates the safety of the trial and suggests appropriate measures if necessary. A detailed description of the interim data monitoring procedure has been specified in a dedicated committee charter.

### Quality control

Several procedures ensure the quality of the trial in compliance with applicable regulatory requirements, GCP and the protocol:

- Written standard operating procedures are implemented
- Personnel involved in conducting the trial are qualified in education, training, and experience. The responsible surgeon (supervising surgeon or operating surgeon) is experienced in the sentinel procedure and axillary dissection (at least 30 cases of sentinel and/or axillary dissection in their career)
- An updated staff list is being kept at the site (template available on the SAKK website)
- Validation of database and statistical analysis
- Quality control principles are implemented
- On-site and central monitoring to evaluate protocol compliance (source data verification, verification of informed consent, etc.) by personnel designated by the SAKK
- Data captured online will be validated in real time, yielding errors (for inacceptable data) and warnings (for possibly inconsistent data – these warnings may be overruled by the user)
- Audit trail of changes
- Medical data review by the coordinating investigator or a delegated person (all CRFs will be reviewed and checked on medical content)
- QA program of RT (facility questionnaire, dummy run, and collection of treatment plans)
- QA program of surgical procedure (specimen radiography performed on all removed lymph nodes after TAS)
- Safety monitoring
- Internal audit procedures
- Central management of deviations and implementation of corrective and preventive measures

### Trial activation procedure

The procedure for trial activation at a site is described in the final protocol letter, which was sent to all sites that committed to participate in the trial. All participating sites must follow the instructions given in this letter for the preparation of site documents. Upon receipt of the site documents, the SAKK CC will submit them to the involved ECs. For foreign sites the instructions are given and the submission to the respective competent authorities is performed by the responsible collaborative group or CRO depending on the country. Any site which is interested in participating in the trial, but has not committed yet, has to contact the SAKK CC first. The investigator will only be allowed to register/randomize patients into the trial after the competent authoritie(s) has/have authorized the trial at the site, the dummy run of the QA-RT has been successful and the SAKK CC has opened the site for accrual.

### Trial termination

The SAKK CC is responsible for submitting the information about trial termination to the Swiss authorities according to the Swiss law (Human Research Act [49]). The SAKK CC will ensure that the information about trial termination is submitted to the respective foreign authorities according to international laws.

### Modifications of the protocol

#### Substantial amendment

This includes any amendment which may have an impact on the conduct of the trial, the potential benefit of the trial, or may affect patient safety, including changes of trial objectives, trial design, patient population, sample sizes, trial procedures, or significant administrative aspects. Such an amendment must be accepted by the SAKK Board and must have the authorization of the respective EC and competent authority (if applicable) prior to implementation.

#### Safety amendment

A safety amendment is a special kind of substantial amendment, which is released when it is necessary to eliminate immediate hazards to trial participants. A safety amendment requires immediate implementation at local sites and is submitted in parallel for authorization to the ECs and the competent authority (if applicable).

#### Non-substantial amendment

Non-substantial amendments, such as minor corrections and/or clarifications that have no effect on the way the trial is conducted, must be submitted to the ECs once a year, together with the submission of the Annual Safety Report. Non-substantial amendments which affect the evaluation of the competent authority must be submitted to the CA as soon as possible.

## Appendix 5: Documentation

### CRFs and reports

CRFs specifically created for this trial are used for documentation. It is very important to adhere to the schedule of visits prescribed in the protocol for all patients. All CRFs needed for the corresponding visit will be displayed automatically in the web-based EDC system for the SAKK trial 23/16. The CRFs must be completed online (www.sakk.ch/edc) in a timely manner. In general, the data should be entered into the CRFs within a month from the visit or medical examination. Sites must use a patient identification list in order to allow identification of a patient. This list must be kept at the site in the investigator’s file.

### Source data

Source data are all information in the original records and certified copies of the original records of clinical findings, observations or other activities in a clinical trial necessary for the reconstruction and evaluation of the trial. Additionally to other source data, the following data are considered to be source data:

- Patient pre-screening log
- Patient screening and enrollment list
- Patient identification list
- QoL questionnaires
- Pathology covering letter

### Record retention

Each study site will retain all essential documents according to ICH GCP. This includes copies of the patient trial records, which are considered as source data, CRFs, patient-informed consent statement, laboratory printouts and all other information collected during the trial. These documents will be stored for at least 15 years after the termination of the trial. The end of this retention period will be communicated to the sites by the SAKK CC. For the patient trial records, which are entered into the EDC system, the sponsor guarantees the access and availability of the data at any time at least 15 years after the termination of the trial. In the event that the principal investigator retires or changes employment, custody of the records may be transferred to another competent person who will accept responsibility for those records. Written notice of such transfer will be given to the SAKK CC. The SAKK will notify the concerned regulatory authorities.

## Appendix 6: Radiotherapy

### General considerations

RT should start preferably within 8 weeks from the last breast surgical procedure and not later than 12 weeks. In case chemotherapy is applied, RT should start within 6 weeks after the end of the last cycle of chemotherapy and not later than 8 weeks.

All patients undergo adjuvant whole-breast irradiation after breast-conserving surgery or chest-wall irradiation after mastectomy. After breast-conserving surgery, a boost to the tumor bed is recommended for all patients of ≤ 50 years of age and considered for patients > 50 years with increased risk of local recurrence (e.g., G3, HER2-pos, triple-negative, accompanying DCIS, LVI, R1/narrow R0) [87–89]. All patients receive regional nodal irradiation, excluding the dissected axilla as a target volume in arm A (levels (II)/III; medial supraclavicular; internal mammary lymph nodes) and including the full axilla (levels I–III; medial supraclavicular; internal mammary lymph nodes) as a target volume in arm B. In case of extensive nodular involvement in arm A, the target volume includes the area at risk of the dissected levels. Inclusion of internal mammary nodes is recommended irrespective of treatment arm [42].

In case of assumed residual or recurrent macroscopic axillary disease after axillary surgery (TAS or ALND) detected by imaging, i.e., performed for RT treatment planning or staging, confirmation with fine-needle aspiration and adjustment of axillary treatment is allowed. The decision whether to apply additional surgery or nodal boost irradiation or irradiation without a nodal boost has to be made interdisciplinary.

### Patient positioning, immobilization, and data acquisition

Patient positioning and immobilization should be performed in accordance with the local operating procedures of the treating center. The position should be consistently reproducible (approximately 5 mm) through the entire course of RT. Patients are positioned supine with the ipsilateral arm or both arms abducted about 120° using a breast board or another form of immobilization to maximize reproducibility. Computer tomography (CT)-based treatment planning is required for all patients irrespective of treatment arms to define the clinical target volume (CTV), the planning target volume (PTV), the nodal levels (n-L1–4, n-IMN, n-interpectoral) and the organs at risk (OAR). The scan thickness should be 2–3 mm and should start at the level of the larynx (superior border) to at least 5 cm below the inferior border of the whole breast target volume always including both entire lungs. This would ensure that there is sufficient CT data for accurate dosimetric calculations. The use of intravenously administered contrast is not mandatory and in accordance with the local operating procedures. The position of the patient is reproduced using skin marks and orthogonal laser beams during treatment preparation and execution.

Treatment using the deep inspiration breath-hold technique is allowed and recommended for left-sided patients and for right-sided patients if essential for the reduction of cardiac dose and ipsilateral lung dose and at the same time ensures sufficient dose coverage.

### Volumes of interest

The CTV, PTV, nodal levels, and OAR must be outlined on all CT slices in which these structures are visible. Standardization of target volume delineation will be generally based on the ESTRO Consensus guideline on target volume delineation [90, 91]. Delineation of all lymph node levels separately and independently from the actual defined nodal CTV will be required for all patients to perform a distinct evaluation of dose distribution within the prospectively planned quality assurance program.

The lymph node levels are in summary:

n-L1(level-I nodes):

Part of the axilla located lateral of the pectoralis minor muscle and more caudally lateral of the thoracic wall. Cranially, the axillary vein should be included with a margin of 5 mm. Laterally, the n-L1 should be limited by a margin of 1 cm around the humeral head (humerus-PRV) in the cranial part and by a line between the lateral edges of the pectoralis major muscle and the deltoid muscle. Caudally, this level ends around the level of costae 4–5 and the dorsal limit is set by the ventral parts of the subscapular and deltoid muscles and more caudally by the ventral edge of the latissimus dorsi muscle.

n-L2 (level-II nodes):

Part of the axilla located dorsal to the minor pectoral muscle. The axillary vein with a 5-mm margin should be embedded including, cranially, the axillary artery. The caudal limit is the caudal border of the minor pectoral muscle. Medially and laterally the volume adjoins level 1 and level III.

n-L3 (level-III nodes):

Volume located medially to the minor pectoral muscle and n-L2. The subclavian vein with a 5-mm margin should be embedded, cranially including the subclavian artery. It is located dorsal the pectoralis minor muscle and ventral to the costae and intercostal muscles. In medial direction the volume is connected to the n-L4.

n-L4 (medial supraclavicular nodes):

This volume represents the supraclavicular volume. Laterally, it connects with the n-L3 including the anterior scalene muscles. Caudally the subclavian vein is embedded with a margin of 5 mm and connects to the n-IMN. The jugular vein is included medially without a margin, excluding the thyroid gland and the common carotid artery. The cranial border is the cranial level of the subclavian artery arch including the subclavian vein with a margin of 5 mm. In case of pathological nodes in level III, the cranial border may be shifted further cranially (maximally below the cricoid cartilage as specified by the Radiation Therapy Oncology Group guidelines).

n-interpect:

These lymph nodes are located ventral to the minor pectoral muscle and dorsal to the major pectoral muscle, while the cranial, caudal, lateral, and medial limits largely reflect the limits of n-L2.

n-IMN (internal mammary nodes):

This volume connects cranially to the n-L4 volume and includes both the internal mammary vein and artery with a 5-mm margin. The dorsal border is the pleura. Caudally, this volume ends from the cranial edge of the fourth rib; in case of a caudally/medially located tumor the cranial side of the fifth rib is allowed to be included.

#### Clinical target volume of breast/chest wall (CTVp_breast/CTVp_chestwall)

The target volume of the breast includes the total glandular breast tissue. The dorsal border of the CTVp_breast is the ventral side of the major pectoral muscle and, where that is not present, the exterior side of the ribs and intercostal muscles. The ventral border is 5 mm under the skin surface. The cranial border extends usually maximally up to the level of the caudal edge of the sterno-clavicular joint. The caudal border is the lowest CT slice with breast shape still visible. The medial border extends maximally to the ipsilateral edge of the sternum. For the lateral border, the breast should be delineated ventral/medial to the lateral thoracic artery. Care should be taken that the CTVp breast encompasses the primary tumor bed.

The boundaries of the CTVp_chestwall are similar to those of the CTVp_breast as described above. Expanders and implants will be included in the CTV. In case of a very thin chest wall, the ventral border of the CTVp_chestwall may be reduced to 2–3 mm under the skin surface for practicability reasons. In case of skin involvement, the ventral border in the respective regions should expand to the skin surface.

#### CTV of the tumor bed boost (CTVp_boost)

The tumor bed should be delineated based on all available information from pre-operative imaging, surgical report, pathology report and the localization of the surgical clips. If oncoplastic surgery was done a close collaboration between surgeon and radiation oncologist is important. The CTVp_boost should include the tumor bed with a margin of 5 mm in all directions to encompass potential microscopic disease extension without exceeding CTVp_breast.

#### CTV of regional nodal irradiation in arm A (CTVn_RNI-A)

The CTV of the regional nodal irradiation in arm A includes the not surgically approached parts of the axilla. This will be in general level III (n-L3), the supraclavicular lymph nodes (n-L4) and the internal mammary lymph nodes (n-IMN). Thus, the final CTV of regional nodal radiation (RNI) should be restricted to include the summation of levels III (n-L3), level IV (n-L4 and the internal mammary lymph nodes (n-IMN). Level II or cranial parts of level II (n-L2) should be included in the nodal CTVn_RNI-A in case the surgical procedure did presumably not include the whole of level II, leaving the lateral and inferior border of the CTV at the discretion of the radiation oncologist. In case clips are used to mark the medio-cranial dissection border of axillary dissection the CTV will be restricted accordingly. There should be no gap between the target volumes of RNI and the resulting target volume of the breast/thoracic wall.

In case of limited nodular involvement (more than three lymph nodes) and biologically low-risk disease (G1- and ER-positive or lateral location and postmenopausal), RNI could be omitted. In case of extensive nodular involvement (e.g., ≥ 50% of removed lymph nodes) and suspected residual disease, the target volume may include the area at risk of the dissected levels. The reason for omitting RNI or including the complete axilla will be queried in the RT CRF.

#### CTV of regional nodal irradiation in arm B (CTVn_RNI-B)

The CTV of the regional nodal irradiation in arm B will be defined like in arm A (n-L3 + n-L4 + n-IMN) but will in addition always include the axillary nodal levels I and II (n-L1 and n-L2).

In case of biologically low-risk disease (G1- and ER-positive and postmenopausal) and lateral tumor location, the irradiation of the internal mammary nodes (n-IMN) may be omitted if the risk of cardiac side effects is considered disproportionately high compared to the benefit of RT to the internal mammary nodes. The reason for omitting internal mammary nodes in the nodal target volume will be queried in the RT CRF. In case of additional complete axillary dissection in arm B target volume will be modified accordingly and spare out the completely approached levels.

#### CTV of nodal boost (CTVn_boost)

In case of assumed residual or recurrent macroscopic disease and decision for a nodal boost without additional surgery, the lymph node tissue visible in imaging and an additional margin of 5 mm should be defined as CTVn_boost.

#### Planning target volume (PTVp_breast/chestwall; PTVn_RNI-A/B)

For the planning target volume (PTV) an additional margin is to be added to the respective CTV in order to take intra-fraction, inter-fraction motion and machine uncertainty into account.

These margins depend on institutional standards. Generally, the minimum of 5 mm CTV-to-PTV expansion is recommended. PTVp_breast/chestwall and PTVn_RNI will be joined to create the PTV, which will be the target volume for treatment planning.

For planning reasons the PTV should be cropped 2–3 mm beneath the skin in case of breast-conserving surgery and 2 mm beneath the skin in case of PMRT. In case of skin involvement the ventral border expands to the skin surface.

#### Organs at risk (OAR)

- Lungs (ipsi- and contralateral)

The lungs should be contoured separately ipsilaterally (Lung_ipsilat) and contralaterally (Lung_contralat) as well as a whole organ as a sum of both (Lung).

- Heart

Superiorly, the whole heart (Heart) starts just inferior to the left pulmonary artery. For simplification, a round structure to include the great vessels as well can be delineated. Inferiorly, the heart blends with the diaphragm. Since cardiac vessels run in the fatty tissue within the pericardium, this should be included in the contours, even if there is no heart muscle visible in that area. The superior vena cava can be included [92].

- Humeral head

The humeral head should be contoured (humeral head) and in addition a planning risk volume including a margin of 1 cm around the humeral head (humeral head_PRV) should be created to help sparing high doses from the humeral head and surrounding tissue by cropping the CTVn_RNI.

- Spinal cord

The spinal cord should be delineated and in addition a planning risk volume including the margin used for the set-up error CTV-PTV expansion should be created (spinal cord_planning risk volume (PRV)).

- Contralateral breast

The contralateral breast should be contoured 5 mm below the skin to the fascia of the pectoralis muscle. The medial border of the organ should end at least 20 mm from the midline including apparent glandular breast tissue.

- Brachial plexus

There is no need to routinely delineate the brachial plexus since keeping the maximal doses of target volumes within the per-protocol constraints accounts for a low risk of brachial plexopathy. However, in case of deviations from these constraints, or if a nodal boost should be performed, the commonly accepted constraints for brachial plexus in the site should be respected.

### External-beam equipment and techniques

Treatment must be delivered on a linear accelerator with a photon beam quality of 4 to 15 MV. Three-dimensional CRT (3D-CRT) techniques or IMRT can be used to configure the irradiation fields. Rotational techniques, such as Tomotherapy®, RapidArc®, IMAT, and VMAT, will also be eligible. For 3D-CRT planning it is recommended to use a technique based on tangential fields with parallel posterior-field edges to cover the PTVp_breast/PTVp_chestwall. For regional nodal irradiation field arrangements are left to the discretion of the investigators to produce an optimal dose distribution for PTV and OAR. Wedges, multileaf collimator (MLC) compensation or field-within-field techniques may be used to optimize dose distribution and compensate for inhomogeneity throughout the PTV.

Treatment using the deep inspiration breath-hold technique is allowed and recommended for left-sided patients and also for right-sided patients if essential for the reduction of cardiac dose and ipsilateral lung dose and at the same time assuring sufficient dose coverage.

The tumor bed boost may be administered using electrons or megavoltage photons at the discretion of the radiation oncologist. Brachytherapy boost is permitted.

### Dose prescription, fractionation, recording, and reporting

The prescribed dose to the breast/thoracic wall as well as the regional nodal pathways is 50 Gy in 25 fractions of 2 Gy or 50.4 Gy in 28 fractions of 1.8 Gy treated on a daily basis, 5 days a week.

Alternatively, a hypofractionated schedule applying 40 Gy in 15 fractions of 2.67 Gy to the same volume is allowed. Treatment centers must define before attending the study which fractionation schedule will be followed throughout the trial to allow for stratification.

As modulated techniques are also permitted in this trial, the definition of volumes and the dose reporting should be in accordance with the ICRU (International Commission on Radiation Units and measurements) report 83 [93]. Dose prescription and normalization will be done to the median dose D50% of the PTV.

Treatment plans will be computed using modern type-B dose calculation algorithms, such as convolution/superposition, Monte Carlo, collapsed cone or equivalent algorithms. Dose calculations will be performed using density heterogeneity corrections.

The boost to the tumor bed if indicated should be delivered sequentially immediately after the completion of whole-breast RT. The fractionation schedule of the boost is left to the discretion of the treating radiation oncologist (e.g., 16 Gy in eight fractions of 2 Gy or 10.7 Gy in five fractions of 2.67 Gy). Mixture of photons and electrons are the preferred modality. Megavoltage photons may be used as single modality if an electron energy of > 12 MeV is necessary to deliver an adequate dose at depth to boost PTV coverage because of the higher skin dose. Details of the boost are left to the discretion of the center and will not be part of the quality assurance of the trial but doses and techniques should be reported.

If indicated, a boost to the lymph node (PTVn_boost) may be delivered sequentially or simultaneously as integrated boost at the discretion of the treating radio-oncologist. A dose of 60 Gy in case of normofractionation is recommended but depends on the individual case.

The use of a skin bolus in case of mastectomy either on the scar or the whole chest wall is left to the discretion of the treating radio-oncologist and will be documented and evaluated within the QA program of the study. If using a bolus the skin dose must follow the constraints for the CTVp_chestwall and the CTV should expand in the area of the bolus to the skin surface.

The goal of treatment planning in both arms is to provide the best possible coverage of the breast/chest wall and nodal PTVs and at the same time minimize inclusion of the heart and lungs. The following dose-volume constraints will be used for dose specification and dose reporting in CTV and OAR (Values in parenthesis refer to the hypofractionated schedule if different from normofractionation) [94].

Target volumes:

**Table:**

Structure	Dosimetric parameter	Per protocol	Minor deviation (acceptable) variation)	Major deviation
CTVp_breast CTVp_chestwall CTVn_RNI-A/B	D_95%_	≥ 95% of the prescribed dose	90–94% of the prescribed dose	< 90% of the prescribed dose
	D_2%_	≤ 107% (≤ 105%) of the prescribed dose	108–110% (106–108%) of the prescribed dose	> 110% (> 108%) of the prescribed dose
	Dmax	≤ 110%	110–115%	> 115%

Organs at risk (OAR):

**Table:**

Structure	Dosimetric parameter	Per protocol	Minor deviation (acceptable) variation)	Major deviation
Lung_ipsilat	V20 Gy (V17 Gy)	< 25%	25–30%	> 30%
	Mean	< 15 Gy (< 13 Gy)	15–18 Gy (13–16 Gy)	> 18 Gy (> 16 Gy)
Lung_total	V5 Gy	< 60%	60–70%	> 70%
Heart	Mean	< 4 Gy	4–6 Gy	> 6 Gy
	V20 Gy (V17 Gy)	< 8%	8–10%	> 10%
	V40 Gy (V35 Gy)	≤ 5%	> 5%	–
Humeral head	D95%	< 50% of prescribed dose	≥ 50% of prescribed dose	–
Spinal cord_PRV	Max	≤ 40 Gy (≤ 33 Gy)	41–45 Gy (34–38 Gy)	> 45 Gy (> 38 Gy)
Breast_contra	Mean	< 6 Gy	≥ 6 Gy	–

Treatment planning will aim to reach the planning aims for the CTV and OAR as defined above with priorities in descending order:

1. Keep CTV and OAR within “per-protocol” limits
2. Keep CTV in “per-protocol” limits and OAR within “acceptable variation”
3. Keep CTV and OAR within “acceptable variation”
4. Keep OAR within “acceptable variation” and CTV coverage as good as possible

Despite the above-mentioned specification of an accepted dose, it is the responsibility of the treating radiation oncologist to achieve as low doses as possible to the organs at risk (see section “Organs at risk (OAR)”) and the medial cervical/mediastinal organs (e.g., thyroid, trachea, esophagus) while achieving sufficient doses to the targets. For organs not mentioned in the table above the constraints commonly used in the site should be applied.

In addition to the dosimetric parameters of the target volumes and the OARs, the DVH parameters will be reported for the nodal levels.

### Treatment-verification schedule

Daily patient set-up should be performed using laser alignment to reference marks on the skin of the patient. Portal images of all fields must be taken during the first treatment session by electronic portal imaging; the use of cone-beam CT is optional. At least the first day set-up verification must be obtained and kept by the treating institution and be available for review upon request. Regular verification by either portal images or cone-beam CT or orthogonal kilovoltage (kV) imaging during the course of treatment is mandatory.

### Quality assurance of radiotherapy

A quality assurance (RT-QA) program will accompany this multicenter trial.

For each participating site, this RT-QA consists of completing the following prior to opening for accrual:

- A facility questionnaire,
- Including an external dosimetry audit (EDA),
- A dummy run

During the trial conduct, the following RT-QA patient-specific procedure must be performed:

- Submission of treatment plans for the first three patients in each treatment arm
- Submission of one randomly selected treatment plan / year and site
- Reporting of relevant treatment parameters for every patient

The “Trial-specific Agreement” explicitly contains a commitment to comply with the QA requirements defined in the protocol.

A dedicated RT-QA website for file download and upload is set up

The facility questionnaire, dummy run, and treatment plans will be reviewed by a group of reviewers under the responsibility of the contact persons for the RT-QA.

#### Facility questionnaire and external dosimetry audit

All sites being opened for accrual must have completed a facility questionnaire that is collecting information about the participating institution and about the methods to be applied for this specific trial. This facility questionnaire can be found at the RT-QA website (see section “RT-QA website and transmission process”) and will preferably be submitted online via the same website. In case of technical difficulties, a paper copy or a CD/DVD-ROM (compact disk/digital versatile disk – read-only memory) may be sent.

All sites being opened for accrual must have a valid EDA (not older than as defined in the applicable national law). This EDA will be submitted online via the RT-QA website. An updated EDA will be submitted at the end of its validity. In case of technical difficulties, a paper copy or a CD/DVD-ROM may be sent.

#### Dummy run

Before opening for accrual, each institution wishing to participate in this trial has to successfully participate in a dummy run. For this purpose, one anonymous case including a CT dataset will be made available. The data exchange will be done in electronic form via the RT-QA website (see section “RT-QA website and transmission process”). The participants will delineate CTV, PTV, and OAR (heart/LADA, lungs, humeral head, spinal cord, contralateral breast) according to the trial protocol. In addition, they will provide a treatment plan fulfilling the requirements defined in the protocol. It will use the fractionation that the institution will use during the trial. The reviewer will evaluate delineation and treatment planning. The results, including eventual corrections, will be communicated back to the institution before its opening for accrual can take place. In case of major deviations, delineation or the treatment plan will be submitted until no major deviations persist.

#### Submission of treatment plans for the first three patients in each treatment arm

Coded treatment plans of the first three patients in each treatment arm will be provided within 1 week after the start of the RT treatment.

The information must include:

- CT data with OAR, nodal levels, CTV, and PTV delineated
- Beam geometry
- Dose distribution
- Dose-volume histogram data for OAR, CTV, and PTV

Patients will be identified by their unique patient number only. It is mandatory for the sites to submit the data to the RT-QA website within 1 week after the start of the RT treatment. The reviewers will check the data and, if necessary, propose changes directly to the QA-responsible radiotherapist. In case of continuously insufficient adherence to the protocol guidelines, the reviewer informs the QA-responsible radiotherapist and the SAKK CC that the next patient of the respective site will be also reviewed, etc. until the plans fulfill the guidelines.

Note: all simulation and portal films and/or digital images will be kept by the institution and only submitted upon request

#### Procedure for other patients

For each site, a RT-QA will be performed randomly for at least one patient / year. The SAKK CC will inform both the QA-responsible radiotherapist at the site and the responsible reviewers which unique patient number was chosen for review. The material must be submitted after the chosen patient has terminated RT. The reviewer will inform the QA-responsible radiotherapist at the site about the outcome of the review. In case of inconsistency or inadequate treatment parameters, additional RT-QAs may be performed.

The QA-responsible radiotherapist is responsible for reporting of treatment-relevant parameters for every patient by completing the RT CRF within 4 weeks after the end of RT.

#### RT-QA website and transmission process

*RT-QA website and transmission of data*

Data must be submitted electronically via a secure web-based file transfer system (https://swiss-dataspace.ch/). This is a server located in Switzerland. Hypertext Transfer Protocol Secure (HTTPS) provides encryption and secure identification of the server. It is a combination of the Hypertext Transfer Protocol with the SSL/TLS (secure sockets layer/transport layer security) protocol. Participating sites will receive a password for access and instructions for data transfer from the reviewer.

*Format used*

RT treatment plans are contained in DICOM-RT files. DICOM files contain medical images (standard for handling, storing, printing, and transmitting information in medical imaging). The RT-specific extensions to the DICOM format contain information including the delineated structures, the treatment plan, and the dose distribution within the body; this format is referred to as DICOM-RT.

*Coding and encryption*

DICOM-RT files contain patient-specific tags that allow identification of the patient. Each tag is stored at a specific address within the files. Before any transmission of DICOM-RT files, coding must be performed by the site. It is the responsibility of the site to ensure that patient name and date of birth will be removed from the DICOM-RT files and be replaced by the unique patient number to enable easy identification within the trial. Identifiers that do not allow patient identification outside the participating institution are permitted (e.g., department-specific patient ID).

Before uploading data to the trial server, the site must code the data and pack them into an encrypted zip-file. Only the respective site and the reviewers have access to these files. In case corrections are necessary, the reviewer will inform the site concerning actions to be taken.

#### Compliance with radiotherapy protocol

Deviations from the RT protocol will be divided in major and minor deviations.

Major deviations:

- D95% < 90% (dose to 95% of volume < 90% of the prescribed dose)
- D2% > 110% of the prescribed dose
- Exceedance of normal tissue dose restriction for major deviation
- Largely inadequate volumes of interest (CTV, PTV, nodal tissue, OAR):

Delineation of specified contouring volumes deviates significantly from protocol guidelines and the protocol-intended volumes are not adequately covered by the prescribed doses

Minor deviations:

- Any deviations not fulfilling the criteria of major deviations
- Inadequate volumes of interest (CTV, PTV, nodal levels, OAR):

Delineation of specified contouring volumes deviates from protocol guidelines but the protocol-intended volumes are adequately covered by the prescribed dose.

## Abbreviations

AE: Adverse event
AJCC: American Joint Committee on Cancer
ALND: Axillary lymph node dissection
BCSS: Breast cancer-specific survival
CRF: Case report form
CT: Computed tomography
CTCAE: Common Terminology Criteria for Adverse Events
CTV: Clinical target volume
DFS: Disease-free survival
EC: Ethics Committee
EDA: External dosimetry audit
EDC: Electronic Data Capture
EudraCT: European Clinical Trials Database
FACT-B: Functional Assessment of Cancer Therapy-Breast
GCP: Good Clinical Practice
Gy: Gray
IBC-RFI: Invasive breast cancer recurrence-free interval
ICH: International Council on Harmonization
ICR: Individual case review
NACT: Neoadjuvant chemotherapy
NCI: National Cancer Institute
OAR: Organs at risk
PET: Positron emission tomography
PMRT: Post-mastectomy radiotherapy
PRV: Planning risk volume
PTV: Planning target volume
QoL: Quality of life
RRR: Regional recurrence rate
RNI: Regional nodal irradiation
RT: Radiotherapy
RT-QA: Radiotherapy quality assurance
SAKK CC: SAKK Coordinating Center
SAKK: Schweizerische Arbeitsgemeinschaft für Klinische Krebsforschung
SLN: Sentinel lymph node
SLNB: Sentinel lymph node biopsy
SOP: Standard operating procedure
SSI: Surgical site infections
TAD: Targeted axillary dissection
TAS: Tailored axillary surgery
UICC: Union for International Cancer Control
WHO: World Health Organization
