# Supplementary material for: Tailored axillary surgery with or without axillary lymph node dissection followed by radiotherapy in patients with clinically node-positive breast cancer (TAXIS): study protocol for a multicenter, randomized phase-III trial
Source: Trials. 2018 Dec 4;19:667. doi: 10.1186/s13063-018-3021-9 (PMC6278139; doi:10.1186/s13063-018-3021-9)
Supplement: Supplementary file 3 — QoL questionnaire. (PDF 514 kb) [file 13063_2018_3021_MOESM3_ESM.pdf]

SWISS GROUP FOR CLINICAL CANCER RESEARCH (SAKK)

SAKK 23/16	QUALITY OF LIFE FORM	FORM QOL (V.1.0, 16.04.2018) Page 1 of 6

PHYSICIAN'S USE:
If the QoL questionnaire was not filled in by the patient, fill in the patients data below and indicate the reason for not filling in:
(1-5) 1) Patient felt too ill to complete the QoL questionnaire at this assessment.
2) Form not presented to patient.
3) Patient refused to participate in this QoL assessment.
4) Patient did not come to clinic.
5) Other, please specify: ....

☐

2316 - _ _ _ _
UPN

Date of completion: __ __/__ __/__ __ __ __
Day Month Year

Nachfolgend finden Sie eine Liste von Aussagen, die von anderen Personen mit Ihrer Krankheit für wichtig befunden wurden. **Bitte geben Sie jeweils an, wie sehr jede der folgenden Aussagen im Laufe der letzten 7 Tage auf Sie zugetroffen hat, indem Sie die entsprechende Zahl ankreuzen.**

	KÖRPERLICHES WOHLBEFINDEN	Überhaupt nicht	Ein wenig	Mäßig	Ziemlich	Sehr
1.	Mir fehlt es an Energie..	0	1	2	3	4
2.	Mir ist übel..	0	1	2	3	4
3.	Wegen meiner körperlichen Verfassung habe ich Schwierigkeiten, den Bedürfnissen meiner Familie gerecht zu werden..	0	1	2	3	4
4.	Ich habe Schmerzen	0	1	2	3	4
5.	Die Nebenwirkungen der Behandlung machen mir zu schaffen	0	1	2	3	4
6.	Ich fühle mich krank	0	1	2	3	4
7.	Ich muss zeitweilig im Bett bleiben	0	1	2	3	4

	VERHÄLTNIS ZU FREUNDEN, BEKANNTEN UND IHRER FAMILIE	Überhaupt nicht	Ein wenig	Mäßig	Ziemlich	Sehr
8.	Ich stehe meinen Freunden nahe	0	1	2	3	4
9.	Ich bekomme seelische Unterstützung von meiner Familie	0	1	2	3	4
10.	Ich bekomme Unterstützung von meinen Freunden	0	1	2	3	4
11.	Meine Familie hat meine Krankheit akzeptiert	0	1	2	3	4
12.	Ich bin damit zufrieden, wie wir innerhalb meiner Familie über meine Krankheit reden..	0	1	2	3	4
13.	Ich fühle mich meinem Partner/meiner Partnerin oder der Person, die mir am nächsten steht, eng verbunden…………	0	1	2	3	4

2316 - _ _ _ _
UPN

Date of completion: __ __/__ __/__ __ __ __
Day Month Year

Beantworten Sie bitte die folgende Frage unabhängig davon, inwieweit Sie zurzeit sexuell aktiv sind. Wenn Sie die Frage lieber nicht beantworten möchten, kreuzen Sie das nebenstehende Kästchen an und fahren Sie mit dem nächsten Abschnitt fort. ☐

	**Überhaupt nicht**	**Ein wenig**	**Mäßig**	**Ziemlich**	**Sehr**
14. Ich bin mit meinem Sexualleben zufrieden	0	1	2	3	4

SEELISCHES WOHLBEFINDEN

15. Ich bin traurig	0	1	2	3	4
16. Ich bin damit zufrieden, wie ich meine Krankheit bewältige	0	1	2	3	4
17. Ich verliere die Hoffnung im Kampf gegen meine Krankheit	0	1	2	3	4
18. Ich bin nervös	0	1	2	3	4
19. Ich mache mir Sorgen über den Tod	0	1	2	3	4
20. Ich mache mir Sorgen, dass sich mein Zustand verschlechtern wird	0	1	2	3	4

**FUNKTIONSFÄHIGKEIT**	**Überhaupt nicht**	**Ein wenig**	**Mäßig**	**Ziemlich**	**Sehr**
21. Ich bin in der Lage zu arbeiten (einschließlich Arbeit zu Hause)	0	1	2	3	4
22. Meine Arbeit (einschließlich Arbeit zu Hause) füllt mich aus	0	1	2	3	4
23. Ich kann mein Leben genießen	0	1	2	3	4
24. Ich habe mich mit meiner Krankheit abgefunden	0	1	2	3	4
25. Ich schlafe gut	0	1	2	3	4
26. Ich kann meine Freizeit genießen	0	1	2	3	4
27. Ich bin derzeit mit meinem Leben zufrieden	0	1	2	3	4

18 June 2012; Copyright 1987, 1997

2316 - _ _ _ _
UPN

Date of completion: __ __/__ __/__ __ __ __
Day Month Year

Bitte geben Sie jeweils an, wie sehr jede der folgenden Aussagen im Laufe der letzten 7 Tage auf Sie zugetroffen hat, indem Sie die entsprechende Zahl ankreuzen.

	**Über-haupt nicht**	**Ein wenig**	**Mäßig**	**Ziem-lich**	**Sehr**
28. Ich leide unter Atemnot..	0	1	2	3	4
29. Die Wahl meiner Kleidung ist für mich eine Belastung.....	0	1	2	3	4
30. Einer meiner Arme, oder beide, sind geschwollen oder empfindlich..……………………………………………	0	1	2	3	4
31. Ich fühle mich körperlich anziehend…………….……	0	1	2	3	4
32. Haarausfall macht mir zu schaffen……………….….	0	1	2	3	4
33. Ich mache mir Sorgen, dass auch andere Familienmitglieder eines Tages dieselbe Krankheit wie ich bekommen könnten..	0	1	2	3	4
34. Ich mache mir Gedanken darüber, welchen Einfluss Stress auf meine Krankheit hat …………………………	0	1	2	3	4
35. Meine Gewichtsveränderung macht mir zu schaffen …	0	1	2	3	4
36. Ich fühle mich als Frau / Ich fühle mich als Mann.............	0	1	2	3	4
37. In einigen Körperbereichen habe ich Schmerzen ………	0	1	2	3	4

Auf welcher Seite hatten Sie die Brustoperation?

Links Rechts (Bitte kreisen Sie eine Antwort ein)

38. Es tut weh, den Arm auf dieser Seite zu bewegen............	0	1	2	3	4
38. Der Bewegungsbereich meines Armes ist auf dieser Seite eingeschränkt...	0	1	2	3	4
40. Der Arm auf dieser Seite fühlt sich taub an.......................	0	1	2	3	4
41. Der Arm auf dieser Seite ist steif…………………………	0	1	2	3	4

Copyright 1987,1997; 03 March 2014

Bitte überprüfen Sie, ob Sie alle Fragen beantwortet haben. Vielen Dank!

2316 - _ _ _ _
UPN

Date of completion: __ __/__ __/__ __ __ __
Day Month Year

Haben Sie eine Schwellung im Bereich des Arms und / oder der Hand?

Nein ☐ Sie müssen die weiteren Fragen **nicht** beantworten.

Ja ☐ Bitte beantworten Sie **alle** weiteren Fragen.

Eine Schwellung im Bereich des Arms und/oder der Hand kann neben physischen und psychischen Folgen auch Einschränkungen in der Ausübung von Aktivitäten mit sich bringen.

Bei jeder Frage müssen Sie **die Zahl umkreisen, die am besten Ihre Situation in den letzten 2 Wochen beschreibt**. Wenn Sie keine Probleme mit der beschriebenen Beschwerde haben, umkreisen Sie die Zahl „0“. Wenn Sie sehr viele Probleme mit der beschriebenen Beschwerde haben, umkreisen Sie die Zahl „10”.

<u>Körperliche Funktionen</u>

Fühlt sich Ihr Arm / Ihre Hand:

	Ganz und gar nicht ↓										Sehr viel ↓
1. Schwer (ermüdet) an?	0	1	2	3	4	5	6	7	8	9	10
2. Steif an?	0	1	2	3	4	5	6	7	8	9	10
3. Geschwollen an?	0	1	2	3	4	5	6	7	8	9	10

Haben Sie am Arm / an der Hand:

	Ganz und gar nicht ↓										Sehr viel ↓
4. Kraftverlust?	0	1	2	3	4	5	6	7	8	9	10
5. Kribbeln?	0	1	2	3	4	5	6	7	8	9	10
6. Schmerzen?	0	1	2	3	4	5	6	7	8	9	10
7. Gespannte Haut?	0	1	2	3	4	5	6	7	8	9	10

2316 - _ _ _ _
UPN

Date of completion: __ __/__ __/__ __ __ __
Day Month Year

Psychisches Befinden

Aufgrund der Probleme am Arm / an der Hand:

Ganz und gar nicht (0) … Sehr viel (10)

8. Fühlen Sie sich traurig? 0 1 2 3 4 5 6 7 8 9 10
9. Fühlen Sie sich entmutigt? 0 1 2 3 4 5 6 7 8 9 10
10. Haben Sie mangelndes Selbstvertrauen? 0 1 2 3 4 5 6 7 8 9 10
11. Fühlen Sie sich angespannt? 0 1 2 3 4 5 6 7 8 9 10

Bei jeder Frage müssen Sie **die Zahl umkreisen, die am besten Ihre Situation in den letzten 2 Wochen beschreibt**. Wenn eine der folgenden Aktivitäten nicht auf Sie zutrifft, markieren Sie den Kreis „nicht zutreffend".

Aktivitäten im Haushalt:

Wie gut können Sie:

Sehr gut (0) … Ganz und gar nicht (10) — ○ nicht zutreffend

12. Putzen (schrubben, staubsaugen, wischen)? 0 1 2 3 4 5 6 7 8 9 10 ○
13. Kochen? 0 1 2 3 4 5 6 7 8 9 10 ○
14. Bügeln? 0 1 2 3 4 5 6 7 8 9 10 ○
15. Im Garten arbeiten? 0 1 2 3 4 5 6 7 8 9 10 ○

nicht zutreffend

Mobilität

Wie gut können Sie:

Sehr gut (0) … Ganz und gar nicht (10) — ○ nicht zutreffend

16. Überkopftätigkeiten (z.B. etwas aufhängen) ausführen? 0 1 2 3 4 5 6 7 8 9 10 ○
17. Schwere Gegenstände aufheben oder tragen (z.B. Eimer Wasser, Einkäufe)? 0 1 2 3 4 5 6 7 8 9 10 ○

nicht zutreffend

2316 - _ _ _ _
UPN

Date of completion: __ __/__ __/__ __ __ __
Day Month Year

Wie gut können Sie:

	Sehr gut ↓ 0	1	2	3	4	5	6	7	8	9	Ganz und gar nicht ↓ 10	nicht zutreffend
18. Auf der betroffenen Seite schlafen?	0	1	2	3	4	5	6	7	8	9	10	○
19. Am Computer arbeiten (>30 Minuten)?	0	1	2	3	4	5	6	7	8	9	10	○
20. Sonnenbaden?	0	1	2	3	4	5	6	7	8	9	10	○
21. Auto fahren?	0	1	2	3	4	5	6	7	8	9	10	○
22. Wandern (>2km)?	0	1	2	3	4	5	6	7	8	9	10	○
23. Fahrrad fahren?	0	1	2	3	4	5	6	7	8	9	10	○

Bedeutende Lebensbereiche und soziales Leben

Wie gut können Sie:

	Sehr gut ↓ 0	1	2	3	4	5	6	7	8	9	Ganz und gar nicht ↓ 10	nicht zutreffend
24. In den Urlaub fahren?	0	1	2	3	4	5	6	7	8	9	10	○
25. Ihre Hobbies ausführen?	0	1	2	3	4	5	6	7	8	9	10	○
26. Sport treiben?	0	1	2	3	4	5	6	7	8	9	10	○
27. Kleidung tragen, die Sie gerne anziehen?	0	1	2	3	4	5	6	7	8	9	10	○
28. Ihren Beruf (bezahlte Tätigkeit) ausüben?	0	1	2	3	4	5	6	7	8	9	10	○
29. Soziale Aktivitäten mit Freunden ausüben (z. B. zu Feiern und Konzerten gehen, essen gehen)?	0	1	2	3	4	5	6	7	8	9	10	○
